# Moving Toward Paperization of Packaging Industry: Use of Laponite and Montmorillonite Nanoclays for Recyclable and Biodegradable High-Barrier Paper

**DOI:** 10.1021/acsomega.5c10619

**Published:** 2026-02-03

**Authors:** Paninga Muiliya, Syeda Shamila Hamdani, Mohamed Shaker, Ian Wyman, Muhammad Rabnawaz

**Affiliations:** † School of Packaging, 3078 Michigan State University, 448 Wilson Road, East Lansing, Michigan 48824-1223, United States; ‡ Department of Chemistry, Michigan State University, East Lansing, Michigan 48824-1223, United States; ⊥ Chemistry Department, Faculty of Science, Tanta University, Tanta, 31527, Egypt

## Abstract

Plastic packaging raises environmental concerns due to the release of microplastics, which has led to increasing interest in paper as an alternative to plastic packaging. However, creating paper that is both recyclable and biodegradable (no microplastic formation over its lifecycle) and still providing the needed oxygen barrier has been a challenging task to achieve. Reported herein is the use of biodegradable poly­(vinyl alcohol) (PVOH) for paper coating, incorporating different nanoclays (laponite and montmorillonite) at various concentrations and assessing their barrier properties. Analysis of the gas, moisture, thermal, and mechanical properties of the samples showed enhanced performance, particularly for laponite-based samples. At a temperature of 23 °C and 50% relative humidity, the oxygen permeability coefficient (OP) of the best sample was 0.875 ± 0.02 cm^3^·mm/m^2^·day, 9-fold lower than that of polyethylene terephthalate (PET) film (11.68 ± 0.41 cm^3^·mm/m^2^·day) of 0.058 mm thickness. The coated paper is also repulpable and recyclable per the FBA protocol. Overall, this work offers an opportunity to develop alternative packaging materials with good oxygen barriers and mechanical properties without releasing microplastics or perfluoroalkyl and polyfluoroalkyl substances (PFAS) into the environment.

## Graphical abstract

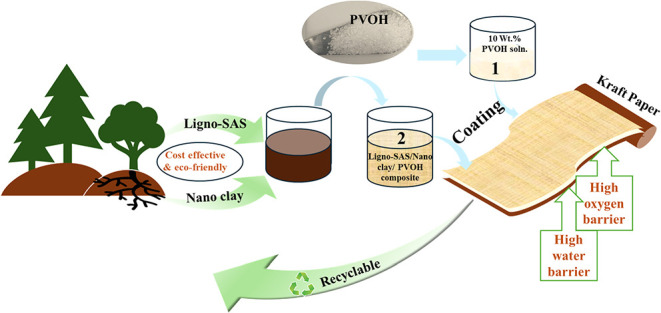

## Introduction

Packaging reduces food and pharmaceutical waste, thereby offering benefits to the environment and society.^1^ However, the packaging industry heavily relies on plastics, whether in the form of single or multilayer materials or plastic-coated paper.^2^ As a result, most commercial products are not environmentally sustainable, and once they enter the environment, they are converted into microplastics, affecting our health and the environment.^3^ Today, microplastics have emerged as a critical health and environmental concern, leading to increased interest in paper packaging.^4^

Paper is a useful material for packaging applications, but it has its own challenges. Despite its renewable and biodegradable nature,^5^ paper is porous and can quickly absorb liquids through mechanisms such as capillary action and through voids in its structure, thus making uncoated paper unsuitable for packaging where plastic-like properties are needed.^6^ Historically, paper is coated with polyethylene,^7,8^ polylactic acid (PLA),^9^ polyhydroxyalkanoates (PHAs), polybutylene succinate (PBS),^10^ waxes,^11^ per- and polyfluoroalkyl substances (PFAS).^12^ However, except for PFAS, all other materials impede paper recycling, as materials such as polyethylene and wax are nonbiodegradable and contribute to microplastic pollution.^13^ PLA is an industry-compostable polymer; it poses environmental risks when it enters oceans because it does not biodegrade quickly in marine environments.^14,15^ Meanwhile, PFAS is known for its environmental impact and potential health risks, including interference with hormones and links to cancer.^16^

There is a strong need for coated paper that performs like plastic but is biodegradable, PFAS-free, recyclable, and that does not make persistent microplastics.^17,18^ Such a paper can be tailored for applications such as disposable single-use items like cups, plates, and food wrappers, which need good water and oil resistance.^19–21^ Other applications need better oxygen barriers for oxygen-sensitive dry products like nuts.^22,23^ Furthermore, another application requires moisture- and oxygen-resistant packaging for products like cookies, dry powdered food items, and chips.

Several studies exist on the use of polymer nanocomposites (PNC), where inorganic nanofillers are incorporated into different polymer matrices. Typical inorganic fillers have included metal oxides,^24,25^ layered silicates,^26^ carbon-based materials (e.g., carbon nanotubes, graphene),^25,27^ and metal-based nanoparticles.^28^ In food packaging applications, layered silicates are mainly used due to their wide abundance, cost, environmental benefits, and surface area.^29^ Research showed that montmorillonite is an excellent and usually preferred nanoclay for PNC coatings in food packaging due to its unique structural and barrier properties, which enable superior gas barrier properties through the tortuous path mechanism.^30^ It is a naturally occurring smectite clay with high aspect ratio, cation exchange capacity, and surface area, which enables its excellent dispersion in polymer matrices, thereby enhancing the barrier and mechanical performance of coated papers.^31,32^ Laponite, which is a synthetic smectite clay with controlled diameter and thickness, has superior structural consistency and purity compared to natural clays.^33^ The negatively charged surface of both clays facilitates strong electrostatic interactions with polymer chains, resulting in uniform dispersion and the prevention of the compromise of barrier properties through agglomeration.^34,35^ Both clays also satisfy safety criteria for direct food contact as they remain immobilized and do not migrate into food simulants once they are incorporated into the polymer matrices.^36,37^

While coatings based on conventional materials like rubber latex, polyethylene, and fluorocarbon exist, research emphasis on PNC has been on using biodegradable and recyclable polymer matrices such as chitosan, polyvinyl alcohol (PVOH), and biodegradable polyesters.^38,39^ Due to its good film-forming ability, biodegradability, gas barrier properties, chemical resistance, and optical properties, PVOH is considered a suitable matrix for PNC coatings in food packaging. Several studies report a reduction in the level of the OTR using montmorillonite, as highlighted by Schiessl et. al.^40^ However, the substrates used are mostly PET, PP films, polyolefins, and other films, which could compromise the recyclability and environmental safety. This work focuses on the application of nanoclays to paper substrates. A summary of some of the recent PVOH-nanoclay studies with paper substrates is shown in Table 1. From the review of the literature, most of these PVOH-nanoclay studies focused mainly on improving the WVTR of the composite paper.^41,42^ In a previous study, we achieved an optimal oxygen barrier by sequentially applying a 5 wt % PVOH bottom layer and a 12.5 wt % zein top layer on a bleached kraft paper at 23 °C and 50% RH.^43^ Under similar conditions, unbleached kraft paper required 20 wt % of zein to achieve its lowest oxygen barrier, which was four times higher than that of the optimal (bleached) sample. In a recent study with unbleached kraft paper, we achieved the lowest oxygen barrier of 262.31 ± 10.66 cm^3^/m^2^·day at 23 °C and 50% RH using 5 wt % starch bottom layer plasticized with 20 wt % glycerol and a 20 wt % zein top protein layer.^44^

**Table 1:** Recent Studies on the Use of PVOH-Nanoclay Composites to Develop High Oxygen Barrier Paper Packaging

system	substrate	optimal OTR	test conditions	refs
PVOH + laponite clay + LignoSAS	Kraft paper	4.145 ± 0.08 cm^3^/m^2^·day	23 °C; 50% RH	this work
PVOH bottom + zein top bilayer	Kraft papers (varied)	Optimal value of 128.0 ± 14.7 cm^3^/m^2^·day on bleached kraft paper	23 °C; 50% RH	^46^
Boric acid cross-linked PVOH	Kraft paper	∼0.89 cm^3^/m^2^·day	23 °C; 0% RH	^47^
PVOH + bentonite nanosheets	Sugar cane paper	264.959 cm^3^/(m^2^·24 h·0.1 MPa)	23 °C, 50% RH for WVTR. Differential method used for OTR (temperature and humidity not specified)	^48^
Waterborne blocked Isocyanate cross-linked starch/PVOH	Kraft paper	Reduced from 13,235.68 to 951.25 cm^3^/(m^2^·24 h·0.1 MPa)	38 °C and 90% RH.	^49^
Oxalic acid modified PVOH cross-linked with Ca^2+^ + acrylic emulsion + CaCO3 nanoparticles	Kraft paper	0.13 cm^3^/m^2^·day (optimal value)	23 °C; 0 RH	^50^

The purpose of this work was to develop more effective and recyclable oxygen barrier paper for the packaging of items that are sensitive to oxygen, such as nuts. To achieve this, we used a suitable oxygen barrier polymer like poly­(vinyl alcohol) (PVOH) and enhanced its performance with nanoclays (laponite and montmorillonite). While other approaches use energy-intensive and time-consuming stabilization materials, this study uses lignosulfonic acid sodium salt (LignoSAS) as a dispersant in the coating dispersions with moderate shearing to isolate clay particles. Unlike many synthetic surfactants, LignoSAS is a byproduct of the pulp process, which is readily available, renewable, cheap, and environmentally friendly.^45^ The use of lignin is also used to ensure that the coated paper is safe and renewable. The performance of the optimal coatings was compared with coatings with a commercial surfactant and commercial films such as poly­(ethylene terephthalate) (PET), which is known for its good oxygen barrier properties. The moisture barrier properties, thicknesses, and mechanical properties of the coated paper were tested, and finally, the coated paper’s repulpability and recyclability were evaluated.

## Experimental Section

### Materials

Blank kraft paper with a basis weight of 135.12 ± 0.07 g/m^2^ was purchased from Uline (WI, USA) and used as a substrate. Lignosulfonic acid sodium salt (LignoSAS, average ∼52,000) and poly­(vinyl alcohol) (PVOH, *M*_w_ 89,000–98,000, 99+% hydrolyzed) were purchased from Aldrich (MA, USA). Laponite clay (Laponite RD - 1) was purchased from TALAS (NY, USA), montmorillonite clay (PGW) was purchased from Nanocor Inc. (IL, USA), and cellulose nanocrystals (CAS: 9004–34–6) were purchased from Cellulose Lab (NB, Canada). Four other clays were purchased from Sigma Aldrich, including surface-modified nanoclay (25–30 wt % trimethyl stearyl ammonium) and montmorillonite (K 10, powder), hydrotalcite, (*M*_w_, 603.98; CAS 11097–59–9), kaolin (CAS 1332–58–7), and nanoclay hydrophilic bentonite (CAS 1302–78–9).

Polyethylene terephthalate (PET) (Laser+ C 9921, F65A) was purchased from Dak America LLC (NC, USA). PET pellets were vacuum-dried at 80 °C for 24 h before being cast into films using a RandCastle RCP-0625 Multi-Layer Cast film extruder at a temperature profile of 250 °C (zone 1 to die), with a chiller (used for cooling) at 90 °C, a screw speed of 19 rpm, with a nip roller at 20 rpm, and with a winding roller at 12 rpm. The thickness of the PET film investigated was 0.058 mm.

### Methods

#### Preparation of PVOH Solution

A 10 wt % PVOH stock solution was prepared by dissolving 10 g of PVOH in 90 mL of deionized (DI) water and heating at 90 °C with stirring until a transparent solution was formed. This solution was then cooled to room temperature before it was applied onto kraft paper.

#### Preparation of Clay Dispersions

Using the formulations identified in Table S1, seven nanoclays (4 wt %, wet basis) were first dispersed in DI water and stirred (450 rpm) in a vial for 3 h to ensure proper dispersion. For the blank samples, the solvents used were DI water and 10 wt % sodium dodecyl sulfate (SDS) solution, respectively. LignoSAS was used as a surfactant in all of the treatment samples. Two representative clays were finally selected for further analysis based on their stability in water, as summarized in Tables S2 and S3. The final coating suspensions were prepared by mixing the clay dispersions with the prepared 10 wt % PVOH stock solution using a vortex mixer, as described in the Paper Coating Procedure.

For comparison, an additional set of clay dispersions was subjected to sonication. After the initial 3 h of magnetic stirring of the clay in deionized water, the dispersion was sonicated for 3 h using an FS30D ultrasonic cleaner (Fisher Scientific) before mixing with the PVOH solution.

#### Paper Coating Procedure

The paper substrates were prepared with the application of PVOH solution onto sheets of kraft paper using a K303 Multicoater (RK Print Coat Instruments Ltd.) multicoater machine. Two sets of base paper were prepared, with one and two layers of PVOH coating, respectively. The coated papers were then air-dried for 24 h at ambient temperature and used as substrates for the nanocoating material.

To investigate the effect of clay dispersions on the paper substrates and at different concentrations, two distinct suspension sets were prepared and applied to the base papers. In the first set, 1 mL of clay dispersion was mixed with 2 mL of the 10 wt % PVOH stock solution in a vial, corresponding to a ratio of 1:2 (v/v). This mixture was homogenized by using a vortex mixer and subsequently coated onto both single-layer and double-layer PVOH-coated papers.

The second set was prepared by mixing 2 mL of the clay dispersion with 2 mL of the 10 wt % PVOH solution, reflecting a 1:1 (v/v) ratio. This mixture was also blended using a vortex mixer to ensure homogeneity before being coated onto another set of single-layer and double-layer PVOH-coated papers.

### Characterization

#### Basis Weight and Coating Load

The basis weight of the paper samples was determined following the ASTM D646 protocol, which measures the mass per unit area of paper (eq 1). The paper samples with dimensions of 2.54 cm × 10.16 cm (1 in. × 4 in.) were preweighed using a digital weighing balance. After the application of the coating material and subsequent drying at room temperature, the samples were weighed again. The coating load of the samples was then calculated as the difference in basis weight before and after the coating process, as shown in eq 1

1 $$\mathrm{basis}\;\mathrm{weight}=\frac{\mathrm{weight}\;\mathrm{of}\;\mathrm{paper}\;\mathrm{speciemen}\;\left(g\right)}{\mathrm{area}\;\mathrm{of}\;\mathrm{paper}\;\mathrm{speciemen}\;\left(m^{2}\right)}$$

#### Thickness

The thickness of all paper samples was measured following the ASTM D645 protocol. Using a digital micrometer (Testing Machine Inc., DE, USA), values of thickness were recorded as the average of 10 different data points taken from each sample.

#### Scanning Electron Microscopy (SEM)

The surface morphology of the nanoclay-coated papers was evaluated using a JEOL 6610 SEM (JEOL Ltd., Japan) system SEM. This was done at an accelerating voltage of 12 kV and at different magnifications. Samples were coated with platinum (with an approximate thickness of 4 nm) in a Quorum Technologies/Electron Microscopy Sciences Q150T turbo pumped sputter coater (Quorum Technologies, East Sussex, England BN8 6BN) under argon purging. The dispersions were spread on a glass slide, and moisture was removed via air drying in a fume hood for 48 h before testing.

#### Energy-Dispersive X-ray Spectroscopy (EDX)

Elemental analysis was performed by EDX using an Oxford Instruments AZtec system (version 3.1; Oxford Instruments, High Wycombe, Bucks, England) equipped with a 20 mm^2^ silicon drift detector (JEOL 6610LV SEM) and an ultrathin window. The paper samples were cut and mounted on aluminum stubs with conductive carbon tabs. Both samples were coated with 12nm of osmium.

#### Transmission Electron Microscopy (TEM)

A JEOL 1400 Flash transmission electron microscope was used to evaluate the quality of the clay dispersions. The clay dispersions were diluted, and a drop of the diluted sample was placed on a Formvar and carbon-coated grid for imaging. The grid was imaged at 100 kV.

#### Dynamic Light Scattering (DLS) Analysis

Particle size distributions were determined by using a Malvern Zetasizer Nano (model ZEN3600) equipped with a 633 nm laser for measurement. Measurements were performed at 25 °C with a temperature equilibration time of 120 s. A backscattering detection angle of 173° was employed. Samples were analyzed in low-volume disposable sizing cuvettes, which were rinsed three times with methanol, followed by three rinses with deionized water before and after each use. Water was used as the dispersant. The refractive index and absorption values of the dispersed material were set to 1.500 and 0.010, respectively. The number and duration of the runs were automatically optimized by the instrument, and the average hydrodynamic diameter was reported.

#### Thermogravimetric Analysis

A TGA550 thermogravimetric analyzer (TA Instruments, New Castle, DE) was used to investigate the thermal stability of the samples. The experiment was performed under nitrogen at a flow rate of 10 mL/min, and the samples were heated from 10 to 600 °C.

#### Differential Scanning Calorimetry

DSC analysis was performed using a TA 2500 analyzer (DE, USA). The analysis was carried out with the temperature range of −50 to 250 °C at the rate of 10 °C/ min. The dispersions of LPD and MCPD were dried in a laboratory oven at 70 °C for 4 h to get rid of all of the moisture.

#### Attenuated Total Reflectance Fourier-Transform Infrared (ATR-FTIR) Analysis

ATR-FTIR (Jasco FTIR-6600 spectrometer, Maryland, USA) spectral analysis was employed to examine the presence of the coating material on the paper samples. Results were investigated for the presence of a particular functional group in the coated paper samples. All spectra were recorded in the wavelength range of 4000–500 cm^–1^ with 32 scans, after 32 background scans.

#### Water Vapor Transmission Rate (WVTR)

A Permatran-W (Model 3/34, Mocon Inc., MN, USA) analyzer was used to measure the WVTR (in units of g/m^2^.day) of the paper samples at 23 °C and a relative humidity of 90%. Sample films were first prepared by using an aluminum foil mask with an opening of 3.14 cm^2^. These films were then mounted in interchangeable cartridges and introduced into the test cells. The dimensions of the paper samples were 4 × 4 cm^2^, and the flow rate of the carrier gas (nitrogen) used was 100 standard cubic centimeters per minute (SCCM). All values were converted into water permeability (WP) (in units of g·mm/m^2^·day) by multiplying the WVTR value by the thickness of the sample.

#### Oxygen Transmission Rate (OTR)

The OTR values of the samples were analyzed by using a Mocon Ox-Tran Model 2/22 analyzer (Mocon, MN, USA). At a relative humidity of 50% and a temperature of 23 °C, samples were analyzed in diffusion cells that separate two chambers (nitrogen and oxygen). As oxygen permeated through the sample films into the nitrogen carrier gas, it was transported to the detector, and the resultant values were expressed in cm^3^/m^2^-day. All values were converted into oxygen permeability (OP, in units of cm^3^·mm/m^2^·day) by multiplying the OTR by the thickness of the sample. Both OP and WP are used here as permeability coefficients, which describe the flow of oxygen and water vapor, respectively, divided by the cross-sectional area of the films at standard pressure.

#### Mechanical Properties

The tensile strength, Young’s modulus, burst strength, and ring crush properties of the samples were measured both in the machine direction (MD) and in the cross-machine direction (CD). Tensile strength and Young’s Modulus measurements were performed according to the TAPPI T494 protocol using a 5565 universal Instron testing machine (Instron, MA, USA). Four paper samples of 25.4 mm (length) × 101.6 mm (width) were prepared using a JDC precision sample cutter and subjected to a load at a speed of 0.5 in/min. The data was obtained as a stress–strain curve.

A TMI crush tester (Model 1210, Instron, MA, USA) was used for the ring crush test (RCT), following the TAPPI T822 standard protocol. From both the CD and MD directions, four paper samples, each with dimensions of 12.7 (length) × 152.4 mm (width), were prepared using a standard cutter. The samples were held in a ring form in a sample holder between two plates of the compression machine. As the lower and driven plate approaches the top and stationary plate, the specimen collapses, and the values of compression strength are then recorded.

Burst strength measurements of the samples were also recorded. Paper samples of the dimensions of 190.5 × 147.32 mm^2^ (7.5 × 5.8 in.) were prepared from both MD and CD directions and analyzed using a Mullen bursting strength tester.

#### Recyclability

The standard used to repulp the paper samples was the “Fiber Box Association (FBA) Voluntary Standard for Repulping and Recycling Corrugated Fiberboard Treated to Improve Its Performance in the Presence of Water and Water Vapor, Part I.” Kraft and nanoclay-coated paper samples were repulped in a modified waring blender and a British disintegrator in water at a pH of 7 (±0.5 pH) and maintained at 125 °F (±10 °F). Each pulped material was then separated with a screen with 0.010 in. slots to determine the fiber recovery as a percentage of the amount of fiber charged. The yield of pulping was calculated as shown in eq 2, with 85% as the required pass value

2 $$\mathrm{yield}\;\%=\frac{\mathrm{Net}\;\mathrm{Accepts}}{\mathrm{Net}\;\mathrm{Accepts}+\mathrm{Net}\;\mathrm{Rejects}}\times 100\%$$

The recyclability test procedure was modified according to the “FBA Voluntary Standard for Repulping and Recycling Corrugated Fiberboard Treated to Improve Its Performance in the Presence of Water and Water Vapor, Part II.” In this process, a 20% coated sample and 80% uncoated base paper were mixed and repulped in a laboratory-scale pulper at pH 7 and 125 °F. The pulped suspension was passed through a vibration flat screen with 0.010 in. slots. Handsheets were then made from screen accepts. Following TAPPI standards, properties, including the coefficient of friction (slide angle, TAPPI T815), short span compression strength (STFI, TAPPI T826), burst strength (TAPPI T403), water drop penetration (TAPPI T831), and stickies counts (TAPPI T277), were investigated and compared to those of a control sample.

## Results and Discussion

The protection and shelf life of food products depend on the permeability of gas molecules through packaging materials.^51^ Nanofillers are used to enhance the package quality as they reduce the diffusion of permeant molecules by creating long tortuous pathways that restrict gas movement through packaging materials.^52^ In this study, 4 wt % formulations of laponite and montmorillonite clay materials were designed using LignoSAS as a dispersant. LignoSAS is a biobased material recovered via sulfite pulping^53^ and is used in food packaging to stabilize emulsions.^54^

The approach employed in this study to prepare the high-barrier paper samples is illustrated in Scheme 1. Seven different clay dispersions made from nanoclay surface modified, montmorillonite, hydrotalcite, kaolin, nanoclay hydrophilic bentonite, laponite, and cellulose nanocrystal were prepared, as shown in Figure S1. Among these, the dispersions containing Laponite and Montmorillonite exhibited superior stability, showing no observable sedimentation. Consequently, these two clays were selected for detailed investigation, and their corresponding dispersions were prepared (Figure S2) by using four different ratios (Tables S2 and S3) between the nanoclays and the LignoSAS surfactant. The final coating materials were prepared by mixing the clay dispersions with 10 wt % of PVOH stock solution (aqueous) in 1:1 and 1:2 ratios (v/v) on stirring with a vortex mixer, and they were subsequently applied onto two sets of paper substrates. The PVOH content was varied to investigate its effect on oxygen barrier properties. The obtained final coating solution was applied onto two sets of paper substrates. The first set of substrates consisted of blank kraft paper (B-KP) coated with a single layer of 10 wt % PVOH and dried at room temperature. For the second set, an additional layer of the same PVOH solution was applied onto the single-layer coated papers. The barrier performance of the resulting samples was then evaluated through WVTR and OTR measurements.

**Scheme 1:**
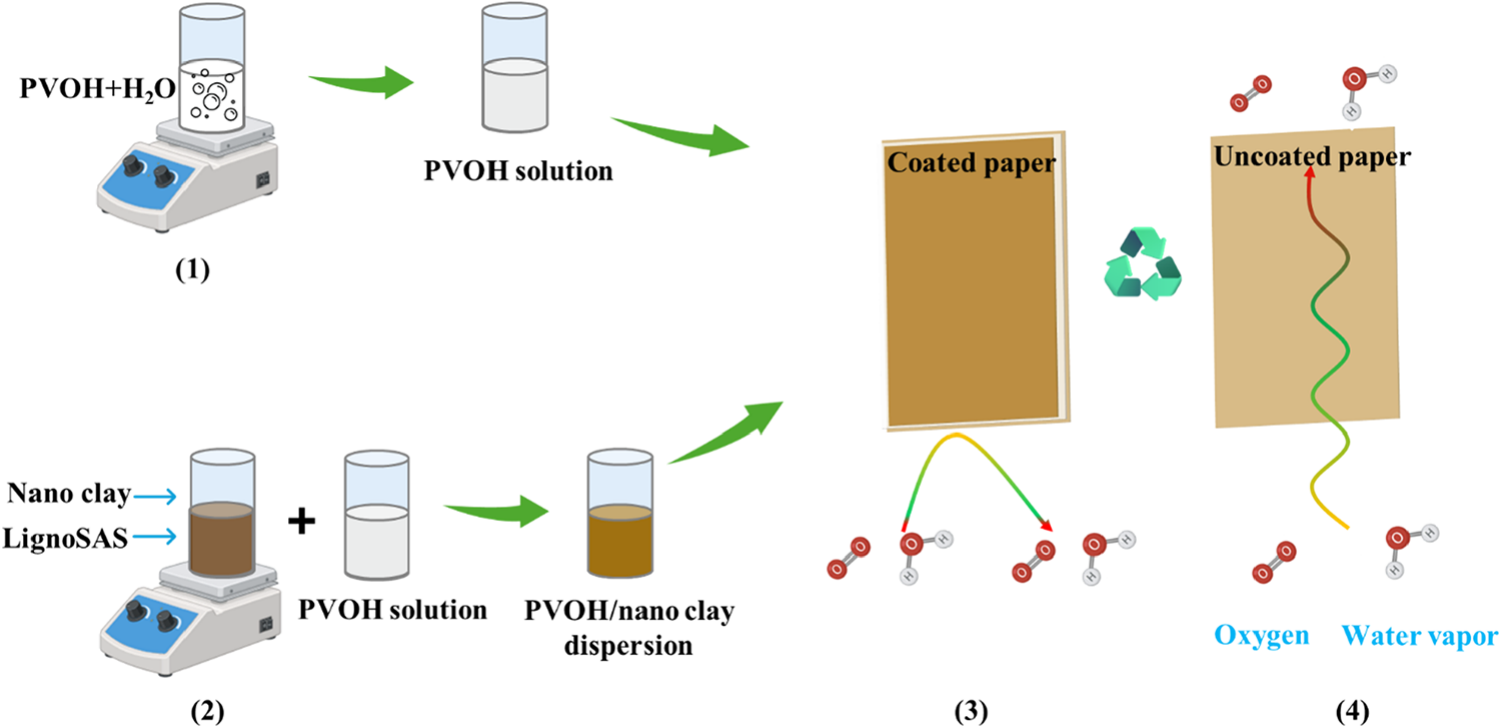
Development of High-Barrier Papers Using Biodegradable Polyvinyl Alcohol, Incorporated with Laponite and Montmorillonite Nanoclays^a^

a Step 1—PVOH was first dissolved (10 wt %) at 90 °C and coated onto kraft papers and left to dry overnight at room temperature. The substrate was obtained by applying a second layer of PVOH to the dried paper until dried. Step 2—Coating dispersions were obtained by mixing the PVOH solution with a pre-prepared dispersion of clays in water and LignoSAS, and then applied on the substrate. Step 3—Barrier measurements were conducted on the dried coated paper. Step 4—The paper was recyclable. Unlike coated paper, the uncoated paper is permeable.

In the preliminary investigations of montmorillonite-coated samples, increasing the ratio of the clay in coatings generally results in a lower OP for most samples across both single- and double-layer systems of the substrates (Tables S4 and S5). Some variations were observed (e.g., in ME–S and MC–D formulations), which indicates the composition of the individual samples may influence their barrier performance. In both single- and double-layer systems, the concentration gradient of the montmorillonite clay in the coatings showed no significant deviations in the WP values of samples (Tables S6 and S7).

Lower OP values were recorded in the laponite-coated paper samples compared with their montmorillonite-coated counterparts. In the first set of substrates (which had a single PVOH layer), increasing concentrations of the clay in each formulation generally led to a corresponding increase in OP, except for the LD–S formulation (the definitions of these formulations, along with their corresponding OTR values, are shown in Table S8). Lower OP was recorded in the double-layer systems, with the least in the LD samples (Table S9). However, no consistent trend was observed in increasing the ratio of clay in the coatings, indicating the influence of the intrinsic sample or material properties on the permeation. Additionally, the difference in concentration of laponite clay in the coating material also had no significant impact on the WP across both single and double-layer systems (Tables S10 and S11). After the OTR and WVTR values of the samples were compared, LPD and MCPD were selected (the formulations of these samples are shown in Table 2). Barrier analyses were conducted on both samples and compared with B-KP, PET film, and the two samples coated with blank formulations (details in Tables S2 and S3).

**Table 2:** Thickness, Basis Weight, and Coating Load Values of Various Paper Samples^a^

sample	nanoclay: LignoSAS (w/w)	nanoclay dispersion: PVOH (w/v)	thickness (μm)	basis weight (g/m^2^)	coating loading (g/m^2^)
B-KP			172.10 ± 1.20	135.12 ± 0.07	0
P-KP			198.50 ± 3.50	164.22 ± 0.88	29.10 ± 0.88
LPA	1:0	1:2	228.60 ± 0.50	172.16 ± 1.81	37.04 ± 1.81
LPB	1:0	1:2	229.90 ± 4.10	172.12 ± 0.34	36.98 ± 0.36
LPD	2:1	1:2	225.76 ± 0.01	167.68 ± 1.13	32.56 ± 1.13
MCPA	1:0	1:2	214.40 ± 2.30	165.84 ± 0.51	30.72 ± 0.51
MCPB	1:0	1:2	213.60 ± 2.70	176.88 ± 0.06	41.76 ± 0.06
MCPD	2:1	1:2	227.60 ± 3.36	176.81 ± 0.21	41.54 ± 0.42

a B-KP = blank kraft paper; P-KP = double-layer PVOH-coated kraft paper; LPA = laponite-coated kraft paper, prepared using a 1:0 ratio of laponite to LignoSAS in DI water; LPB = laponite-coated kraft paper, prepared using a 1:0 ratio of laponite to LignoSAS in 10 wt % SDS; LPD = laponite-coated kraft paper, prepared using a 2:1 ratio of laponite to LignoSAS in DI water; MCPA = montmorillonite-coated kraft paper, prepared using a 1:0 ratio of montmorillonite to LignoSAS in DI water; MCPB = montmorillonite-coated kraft paper, prepared using a 1:0 ratio of montmorillonite to LignoSAS in 10 wt % SDS; MCPD = montmorillonite-coated kraft paper, prepared using a 2:1 ratio of montmorillonite clay in DI water.

### Thickness, Basis Weight, and Coating Load

The thicknesses, basis weights, and coating loads of the paper samples investigated are shown in Table 2. The coating loads ranged from 32.56 to 37.04 g/m^2^ for the laponite-coated paper samples and from 30.72 to 41.76 g/m^2^ for the montmorillonite-coated paper samples. The montmorillonite-coated paper sample, MCPD, was observed to have a higher coating load than the laponite-coated paper sample, LPD.

### ATR-FTIR Analysis

The FTIR spectra (Figure 1) of the paper samples and nanoclays were measured and compared with those of the two reference samples (blank kraft and PVOH-coated paper). Major absorption bands observed in the blank kraft paper were characteristic of polysaccharides, including peaks appearing around 3300 cm^–1^ (O–H stretch), 2900 cm^–1^ (C–H stretch), and 1000 cm^–1^ (C–O–C stretch).^55^ The signature vibrations from PVOH and blank kraft paper were equally observed on the PVOH-coated paper. Both laponite and montmorillonite are phyllosilicate minerals with a characteristic Si–O stretching band (around 1000 cm^–1^) and an O–H stretching band corresponding to the lattice’s hydroxyl group (3450 cm^–1^).^56^ When contrasted with P-KP, the IR spectrum of the laponite-coated paper sample showed IR stretches from OH, C–H, C–O–C, and Si–O groups, confirming the presence and stability of the coating material on the paper. Similarly, the presence of the montmorillonite coating material on the paper was confirmed by signature bands on the nanoclay.

**Figure 1:**
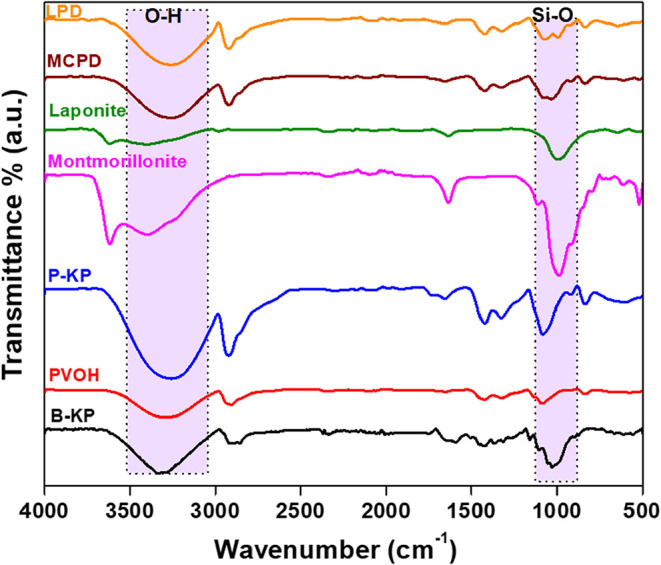
ATR-FTIR spectra of the solid coating material (laponite, montmorillonite, PVOH), nanoclay-coated paper samples (LPD, MCPD), and control papers (kraft paper, PVOH-coated paper).

### Energy-Dispersive X-ray Spectroscopy

Energy-dispersive X-ray (EDX) spectra of LPD and MCPD samples have been established, as illustrated by Figure 2. The spectra of both samples are dominated by carbon and oxygen, which is characteristic of cellulose paper and the organic polymer coating. Typical of synthetic hectorites like laponite, LPD has a higher percentage of magnesium with no aluminum detected, which is attributed to its composition and the isomorphous substitution in the crystal lattice.^57^ It also has a higher composition of carbon and a lower percentage of oxygen, which could be suggestive of less oxygen-rich groups like silicates and hydroxyls compared to MCPD. These variations confirm the incorporation of the nanoclays consistent with their formulations. For instance, MCPD with higher oxygen-rich groups is suggestive of a higher nanoclay content or clay-polymer interaction, which could potentially provide a more tortuous pathway and increased mechanical strength of the paper.

**Figure 2:**
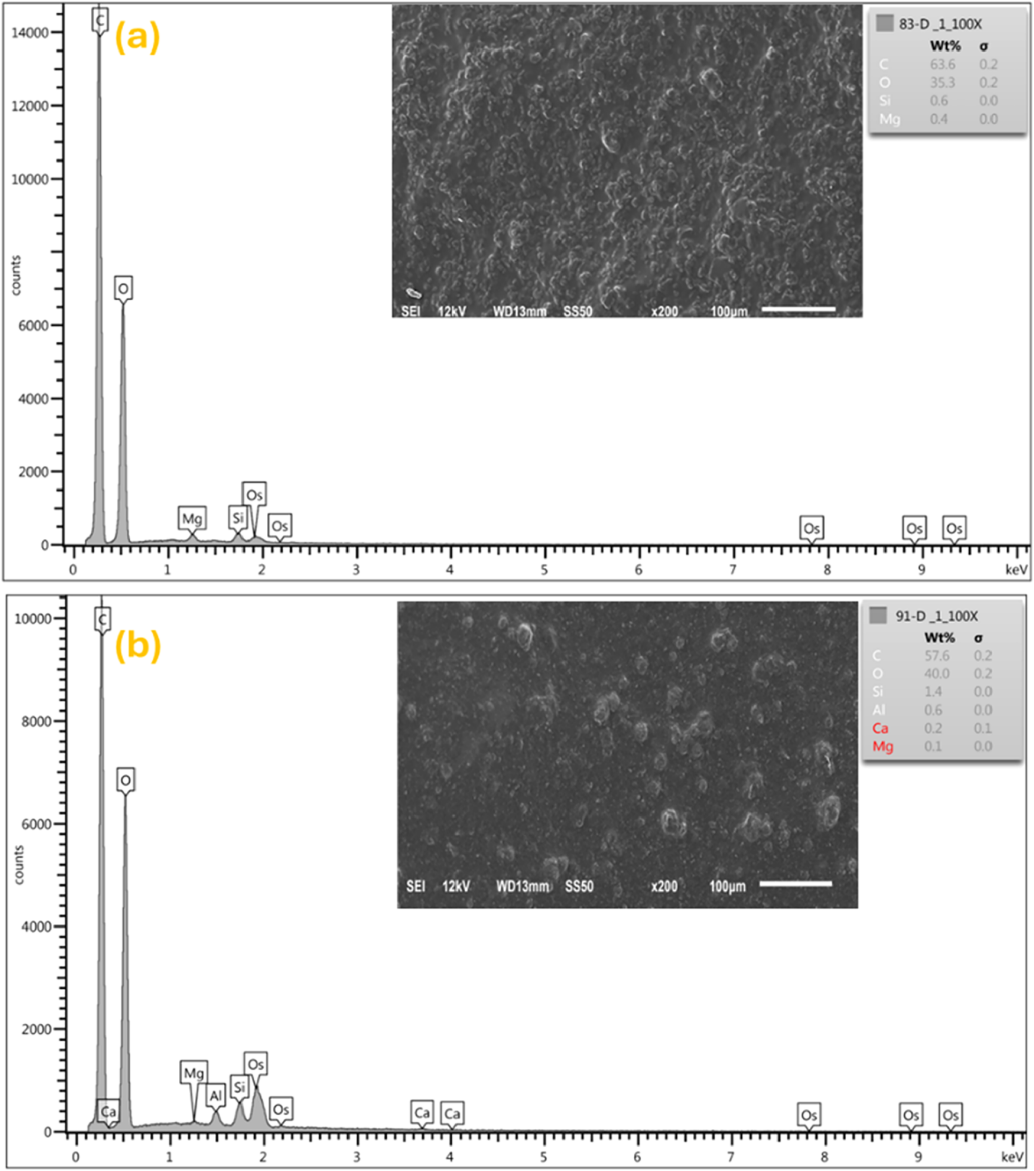
EDX spectra of (a) LPD paper coated with the laponite clay dispersion and (b) MCPD paper coated with a montmorillonite dispersion.

### Thermogravimetric Analysis

To determine the thermal stability of our samples, thermogravimetric analysis (TGA) was employed for LPD and MCPD as well as control samples (B-KP and P-KP). Figure S3a illustrates the thermographic behavior of samples, where a minor weight loss is seen below 100 °C for all samples, which is attributed to the release of physically attached moisture. The principal degradation occurs between 250 and 400 °C, representing the breakdown of the polymer structure. PVOH shows the earliest thermal decay, starting around 250 °C, and its mass decreases sharply, leaving less than 5% residue by 500 °C. This outcome reflects its comparatively low heat resistance and poor char-forming ability. In contrast, paper samples such as B-KP, P-KP, LPD, and MCPD begin to decompose at slightly higher temperatures (around 280–300 °C) and lose weight more gradually, with the main loss centered around 330–360 °C. These samples preserve roughly 15–20% of their initial mass at 600 °C, suggesting the formation of thermally stable char residues. The higher char yield and slower degradation of the modified systems (P-KP, LPD, MCPD) relative to neat PVOH demonstrate that lignocellulosic incorporation and clay modification improve the thermal stability and structural robustness of the materials.

The thermal characteristics of the paper samples are also supported by DTG curves (Figure S3b). The first and second degradation peaks of PVOH at around 250 and 350 °C are attributed to dehydration and the oxidative breakdown of the residual amorphous carbon. P-KP, LPD, and MCPD displayed a sharp and single maximum at around 360–380 °C. Their relative similarities, as also observed in the TGA report, suggest comparable degradation pathways dominated by the pyrolysis of polysaccharides. The maximum decomposition rate was slightly higher for P-KP (1.229%/°C), indicating a faster mass-loss process. MCPD and LPD, with heterogeneous compositions, showed lower maximum degradation rates of 1.090%/ °C and 1.081%/ °C, respectively. In general, the DTG results are consistent with the TGA curves, stating that coated papers have higher thermal stability.

### Water Permeability Analysis

The water vapor permeabilities (WP) of the paper samples were measured at a relative humidity of 90% and a temperature of 23 °C. Four samples were used as controls, including blank kraft paper (B-KP), double-layer PVOH paper (P-KP), and paper coated with the two blank (LA/MA and LB/MB, described in Tables S1 and S2) formulations.

In the laponite formulations, the application of the nanocoating material prepared with 10 wt % SDS (LPB) led to a decrease in the WP value by 40%, 48%, and 70% in comparison to B-KP, P-KP, and the DI-water-based blank sample (LPA), respectively (Figure 3a). SDS, which is commonly used as an emulsifier,^58,59^ improved the water vapor permeability of the LPB-control sample by dispersing the coating material and filling the pores on the paper, as seen in the SEM image of the sample (Figure S4). The paper sample coated with LignoSAS-emulsified laponite formulation (LPD) showed an approximately 60% decrease in its WP value compared to that of B-KP. It also showed a lower WP value than the other control samples, except for the SDS-based blank. Meanwhile, the PET film showed better and far lower WP than the two paper samples that were coated with LignoSAS-emulsified formulations (LPD and MCPD).

**Figure 3:**
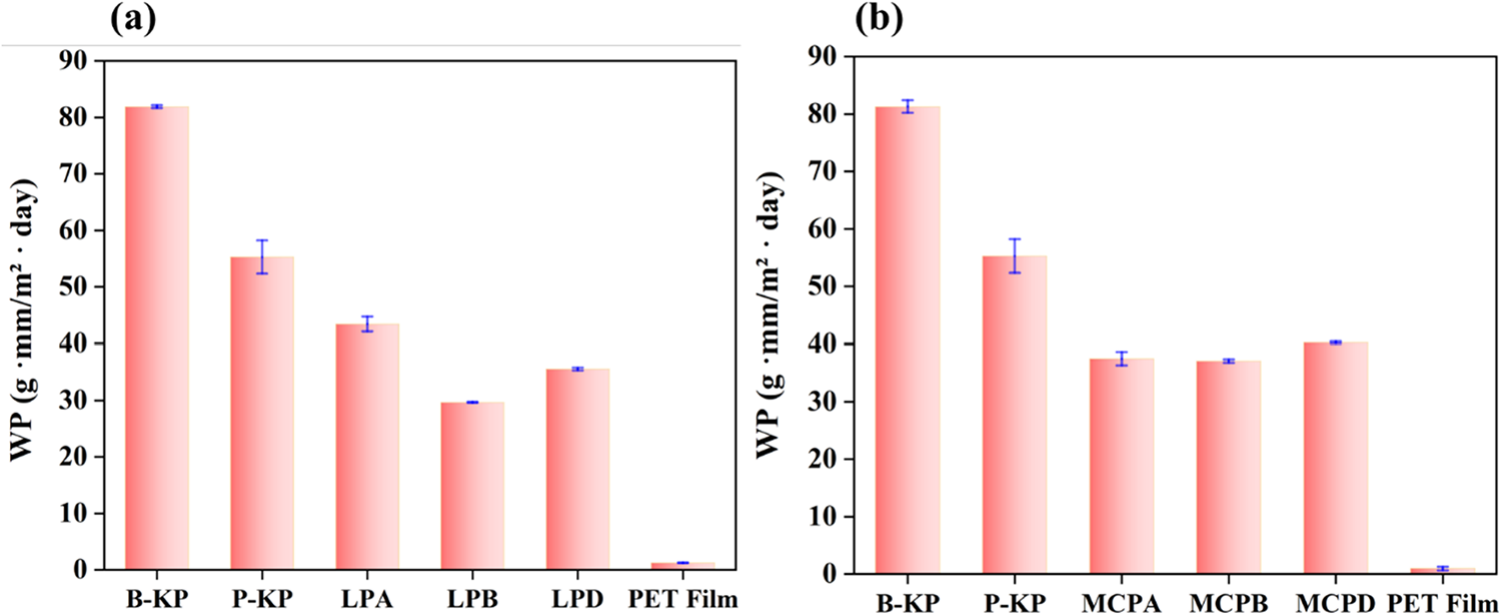
Water vapor permeabilities of paper samples coated with nanoclays compared with those of blank kraft paper (B-KP), PVOH-coated paper (P-KP), and PET film. All measurements were recorded at the temperature and relative humidity of 23 °C and 90%, respectively.

In contrast, the WP value of the blank SDS-based paper coated with montmorillonite clay has comparable values to the DI water-based blank paper sample (Figure 3b). The WP value of the LignoSAS-emulsified paper sample, MCPD, decreased by 30% and 50% in comparison to P-KP and B-KP, respectively. However, with respect to the blank samples (MCPA and MCPB), the WP of MCPD increased by about 7% and 11%, respectively. As seen in the SEM images, this increase in WP could be a result of the rough surface morphology of the MCPD sample, which creates pathways that facilitate the diffusion of water vapor through the coating material and coated paper. Additionally, the MCPD sample showed a much higher WP (40.28 ± 0.160 g·mm/m^2^·day) than that of the PET film (0.98 ± 0.33 g·mm/m^2^·day)

### Oxygen Permeability Analysis

The oxygen permeability (OP) of the paper samples was measured at a relative humidity of 50% and a temperature of 23 °C. LPB showed an OP value that was about 98% lower than that of LPA (Figure 4a), which could be attributed to the uniform clay dispersion and filling on the substrate, as seen in Figure S4. LPD and the PVOH-coated controls have low and similar OP values of 0.88 ± 0.02 cm^3^·mm/m^2^·day and 0.92 ± 0.26 cm^3^·mm/m^2^·day, respectively. LPD exhibited an OP value that is about 93% lower than PET. The OP value of the blank kraft paper was beyond the Ox-Tran analyzer’s saturation point (≥3184.7 cm^3^/m^2^-day) for the area of the hole (3.14 cm^2^) used in the sample film.

**Figure 4:**
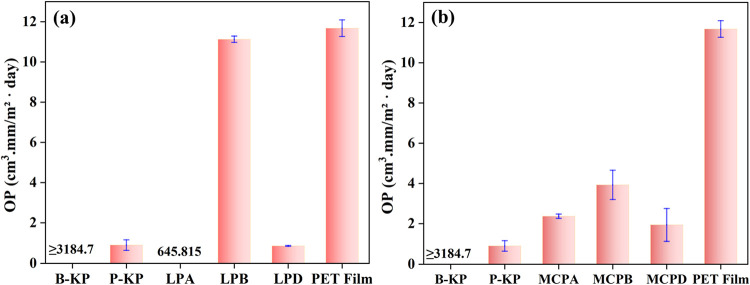
Oxygen permeabilities of laponite-coated paper samples (a), and montmorillonite-coated samples (b), compared with unbleached kraft paper (B-KP), PVOH-coated paper (P-KP), and PET film. All measurements were recorded at the temperature and relative humidity of 23 °C and 50%, respectively.

On the other hand, compared to the DI-water-based montmorillonite blank formulation, using SDS as an emulsifier in sample MCPB led to an increase in the OP value by about 50% (Figure 4b). The OP value of MCPB also increased significantly (0.92 ± 0.26 to 3.94 ± 0.73) compared to the P-KP sample. However, on the application of the coating material with 50% surfactant (MCPD), the OP value decreased from the mean value of 3.94 ± 0.73 cm^3^·mm/m^2^·day (for MCPB) to 1.95 ± 0.81 (for MCPD). When compared with PET film, MCPD showed about an 82% reduction in its OP value.

It was observed that PVOH-coated (P-KP) paper showed a low and comparable OP value to those of LPD and MCPD. We attribute this to the more uniform barrier of the PVOH coating and the concentration of clay formulation used in the study. The continuous and uniform barrier in P-KP could be due to the stacking effect of the two layers, the crystalline structure of PVOH, and the strong intermolecular hydrogen bonds along the PVOH polymer chains.^60^ On the other hand, polymer nanocomposites have been reported to display much better barrier properties, mostly at 1–3 wt %.^60–62^ Since we have used a 4 wt % nanofiller concentration in this study, this could be responsible for the similar and low (in the case of MCPD) OP values of the LPD and MCPD samples with respect to the PVOH-coated paper sample.

### Mechanical Properties

The mechanical properties of cellulose-based materials are important factors in determining their suitability for industrial applications. Our samples were analyzed to investigate the impact of nanocoating materials on mechanical properties such as the tensile strength, Young’s modulus, elongation at break, burst strength, and ring crush resistance (Figure 5). In the machine direction, LPD showed a slight increase (almost negligble) in tensile strength (43.0 ± 0.50 MPa) compared to the uncoated kraft paper (42.6 ± 0.42 MPa) and the SDS-based blank sample (41.25 ± 0.07 MPa), and a decrease in its tensile strength compared to LPA and the PVOH-coated paper (P-KP). LPD also showed a slight increase in tensile strength in the cross direction compared to all of the control samples. Similar trends were also observed in the cross and machine directions of the MCPD sample. In the cross direction, its tensile strength (21.95 ± 0.77 MPa) was slightly higher than that of MCPB (21.55 ± 0.21 MPa) and lower than that of the uncoated kraft paper (22.25 ± 0.35 MPa). In the machine direction, it was observed to have a higher value than those measured in MCPB and the uncoated kraft paper, and a lower value compared to the P-KP sample.

**Figure 5:**
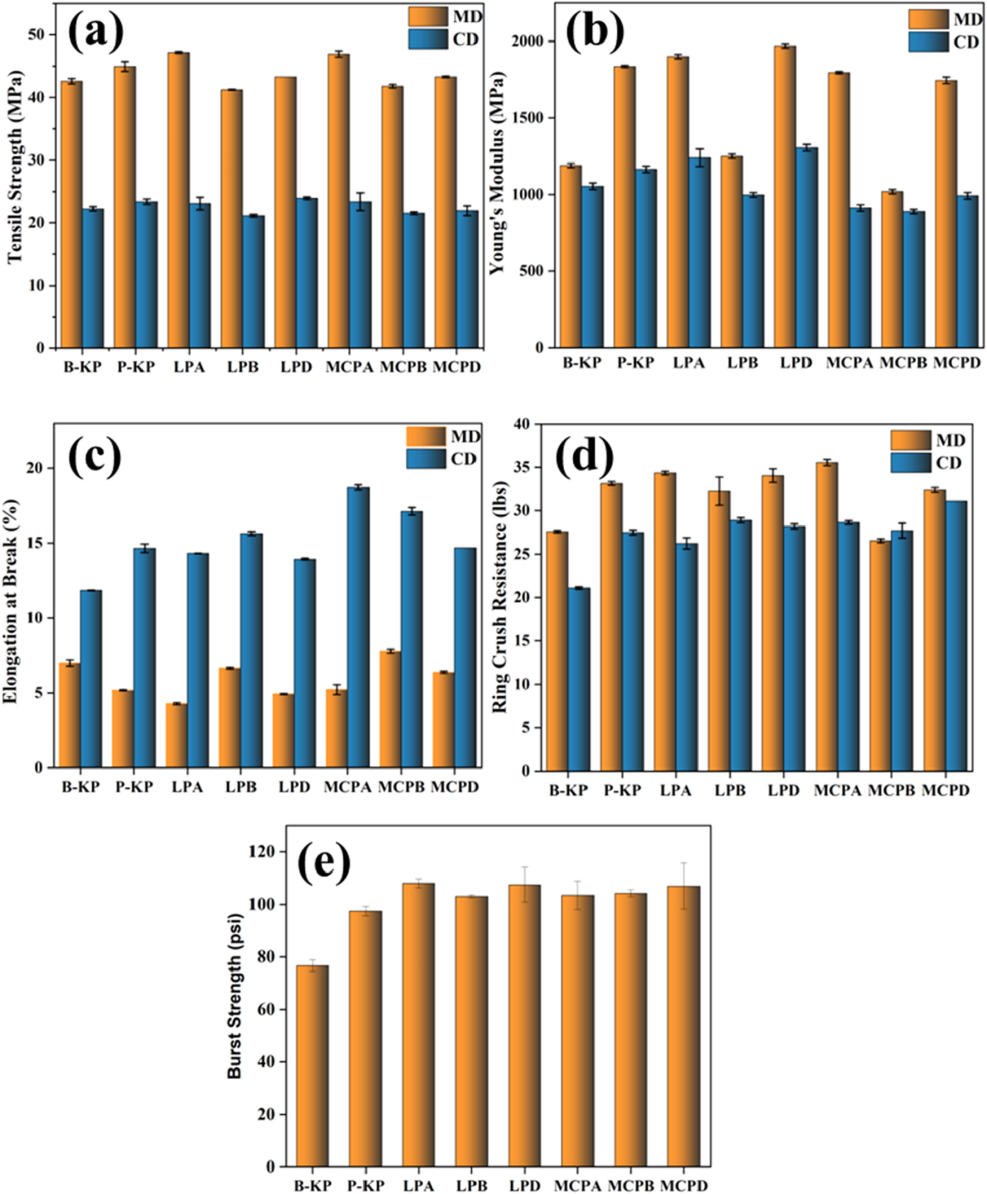
Mechanical tests of paper samples, including (a) tensile strength, (b) Young’s modulus, (c) elongation at break, (d) ring crush resistance, and (e) burst strength.

Regarding the Young’s modulus data for the laponite-coated samples, LPD exhibited a higher value (1970 ± 14 MPa) than those of the kraft paper (B-KP), PVOH-coated paper (P-KP), and LPB samples in both the machine and cross directions, which is an indication of its higher stiffness. In the machine direction of the montmorillonite-coated samples, MCPD had a higher value (1745 ± 21 MPa) than that of MCPB and B-KP, but lower than that of the MCPA and P-KP controls. In the cross direction, the Young’s modulus of MCPD is also higher than those of MCPB and MCPA, but slightly lower than that of B-KP.

The elongation at break (EB) data also reveal the influence of the coating materials on the flexibility of the paper. Both LPD and MCPD exhibited higher EB values than their corresponding DI-water-based blank samples but slightly lower values than the SDS-based blank samples. P-KP had a lower EB value than that of MCPD, but it was higher than that of LPD.

In the cross direction, RCT data reveal that the MCPD and LPD samples possessed increased ring crush resistance compared to the uncoated kraft paper. Very little deviation is seen in their RCT values when compared to other controls, except for MCPD, which has a higher RCT value (32.4 ± 0.3 lbs) than that of MCPB (26.6 ± 0.2 lbs). This increase is attributed to the reinforcing properties of the nanoclays, which impart strain and stiffness to the samples.^63^ Although the samples MCPD and LPD displayed slightly lower but similar values compared to those of their respective control samples in the machine direction, these samples exhibited a higher average ring crush resistance.

From the burst test results, the baseline burst strength of B-KP was observed to be 76.7 ± 2.2 psi. Application of both the laponite and montmorillonite coating material remarkably increased the baseline value, indicating that the paper samples had a good resistance to rupture under pressure. LPD and MCPD showed high burst values of 103.1 ± 0.2 and 107.5 ± 7 psi, respectively.

### SEM Analysis

The SEM analysis provided detailed insights into the dispersion and uniformity of the coatings, which are critical factors influencing water vapor permeability (WP) and oxygen permeability (OP). The neat dispersions (without PVOH) and those obtained after adding PVOH were characterized, and the distributed clay particle sizes were recorded, as shown in Figure 6. Laponite clay was uniformly dispersed in deionized water, with particle sizes in the range of 0.68–2.22 μm (Figure 6a). Montmorillonite clay dispersed in deionized water showed some degree of aggregation but retained a structured appearance (Figure 6c). SEM images of the polymer-clay coatings (after addition of PVOH) (Figure 6b,d) showed rough structures, with more aggregation seen in the montmorillonite dispersion. We attribute this effect to incomplete exfoliation^64^ of the nanolayers in the matrix, possibly due to the vortex mixing technique used in this study. The size of aggregated particles in laponite dispersion was in the range of 0.46–4.27 μm, while the montmorillonite counterpart exhibited a more irregular morphology with particle size in the range of 0.53–2.59 μm.

**Figure 6:**
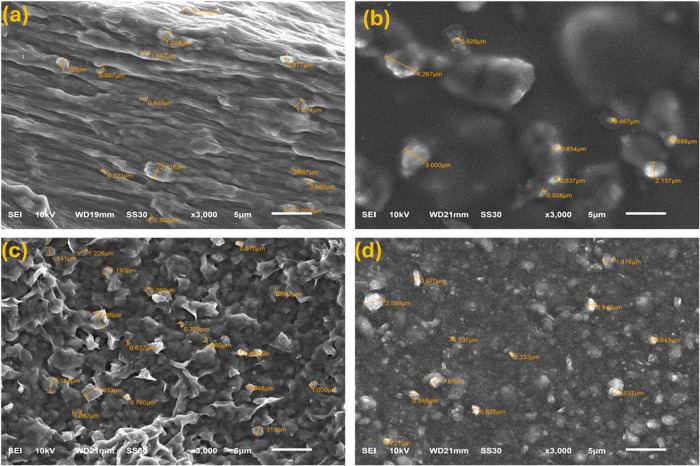
SEM Images of (a) the laponite dispersion (before mixing with PVOH), (b) the laponite dispersion (after mixing with PVOH), (c) the montmorillonite dispersion (before mixing with PVOH), and (d) the montmorillonite dispersion (after mixing with PVOH).

The coating material was applied to kraft paper, and SEM images were recorded to observe the surface morphologies of the resultant coated papers. By comparing samples prepared with and without surfactants, we aimed to elucidate the role of surface roughness and particle distribution in the barrier properties of the coated papers. As reported in Figure 7a, B-KP showed a fibrous and irregular structure, having many holes and voids, while the P-KP showed a smoother and more homogeneous surface, indicating the uniform coverage of all of the pores (Figure 7b).

**Figure 7:**
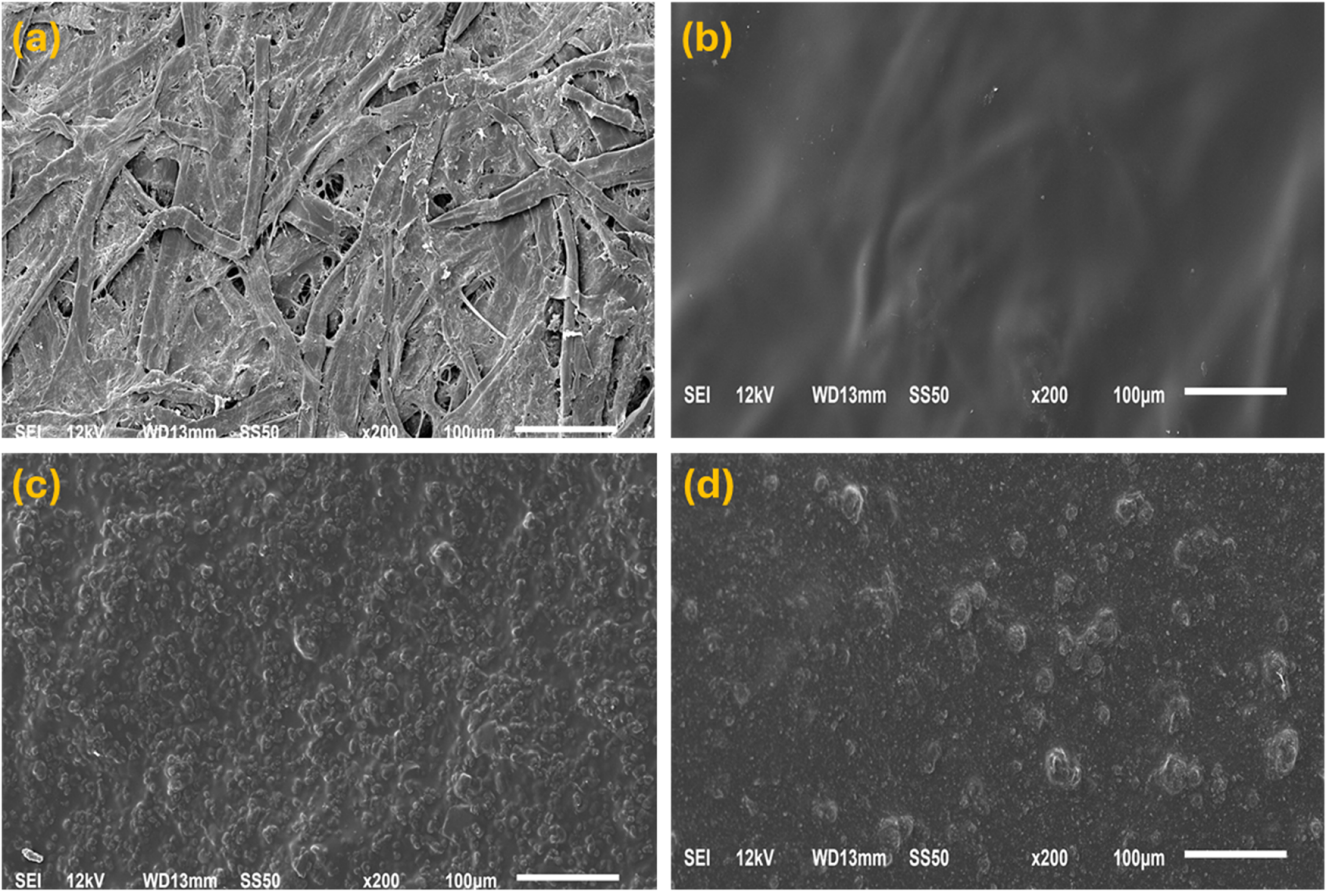
SEM images of (a) blank kraft paper, (b) double-layer-coated PVOH paper, (c) LPD, and (d) MCPD.

The paper coated with a laponite clay material possessed an evenly dispersed particulate layer (Figure 7c). Conversely, the paper coated with montmorillonite clay (Figure 7d) displayed a surface with larger and more aggregated clay particles. This variation in exfoliation and surface morphologies validates the higher WP and OP values of the montmorillonite clay-based sample (MCPD), as compared with the laponite clay-based sample (LPD).

We also evaluated the effect of sonication on the particle size, coating morphology, and barrier properties of the resulting coated papers. For the best-performing samples, LPD and MCPD dispersions were sonicated for 3 h. TEM, SEM, and barrier analyses were performed on the sonicated dispersions and compared with the original and nonsonicated samples, with detailed results provided in the Supporting Information (pages S16–S20). Overall, sonication had little to no positive effect on the barrier properties of the paper samples.

### TEM Analysis

The quality of the laponite and montmorillonite dispersion was evaluated by using TEM, as reported in Figure S5. Like the SEM data, moderate exfoliation of clay particles (dark lines) was observed in the laponite coating, which is a resultant effect of moderate dispersion in the polymer matrix (in white). On the other hand, a greater agglomeration of clay particles was observed in the montmorillonite coating.

### Dynamic Light Scattering (DLS) Analysis

To further analyze the quality of the dispersion, DLS analysis was performed on the dispersions, including clay in water and clay dispersed in lignosulfonate acid sodium salt (LignoSAS), as reported in Figure 8.

**Figure 8:**
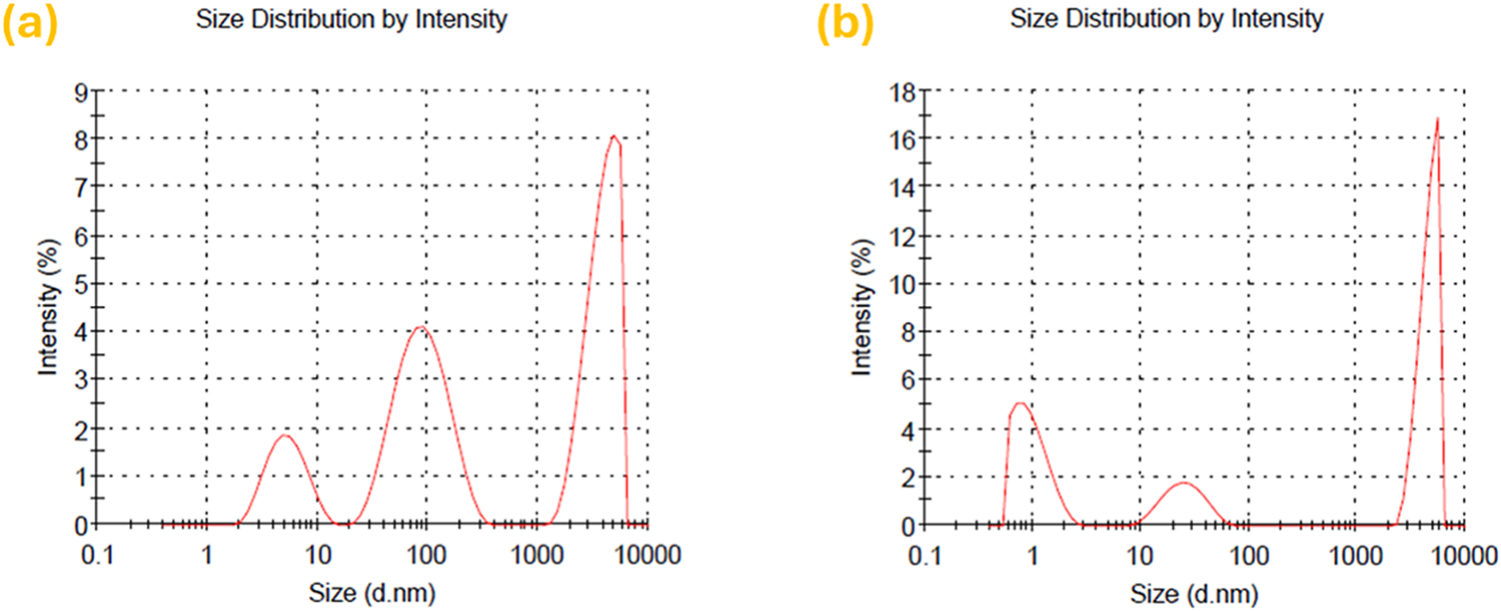
DLS report of (a) laponite dispersion in water (without LignoSAS) and (b) laponite dispersion in water (with LignoSAS dispersant).

The intensity-weighted distribution exhibits three modes around 10 nm, 100 nm, and 10 μm, typical of partially exfoliated clays containing individual platelets, small tactoids, and larger aggregates.^65^ The smallest mode corresponds to single or few-layer platelets, the intermediate mode reflects tactoids formed by incomplete delamination, while the dominant micrometer-scale peak corresponds to a fraction of aggregates with a large scattering cross-section that amplifies their intensity.^66^ These findings are consistent with the SEM and TEM observations, which reveal nonuniform dispersion of clay particles within the polymer matrix.

### Recyclability

Although multilayer laminates have high barrier properties, their use in food packaging poses major challenges, such as the difficulty of separating the layers, which limits their recyclability.^67^ This makes the recyclability testing of our paper samples a very important aspect of this study. According to TAPPI standards, the LPD and MCPD samples were tested under the conditions described in the recyclability subsection of the Experimental Section. Kraft paper (base paper) was also screened using identical conditions and used as a control sample. The result indicated that the tested samples, LPD and MCPD, passed the repulping test with 98.64% and 98.61% fiber recovery (Table 3), exceeding the minimum requirement of 85%. The screen accepts and screen rejects of tested samples are shown in Figure S6.

**Table 3:** Percentage Yield of the Repulping Process for Samples LPD and MCPD

		sample code	
content	unit	LPD	MCPD
Moisture	wt %	7.01	6.90
Sample charged	g	23.40	26.73
Screen rejects	g	0.19	0.23
Screen accepts	g	13.78	16.36
Yield of the sample	wt %	98.64	98.61
Pass/Fail		Pass	Pass

The samples were further tested for their recyclability. The accepted pulp was used to make handsheets, which were subjected to various tests in contrast to the handsheet obtained from the pulp of the base paper. The various tests that were conducted included stickies count, water drop penetration, slide angle, burst strength, and short span compression strength measurements. The results showed that there was no significant change in the handsheets for the tested recycled paper samples in comparison to the base paper. Changes in the aforementioned properties observed in the tested samples were less than 15% and no stickies were observed (Figure S7). General data showing all of the properties of recycled paper are shown in Table 4.

**Table 4:** General Properties Studied to Evaluate the Recyclability of Paper Samples LPD and MCPD

performance	unit	base paper (B-KP)	LPD	MCPD
Basis weight	g/m^2^	115	108	105
Coefficient of Friction	Degrees (°)	35.8	38.2	36.9
Water drop penetration	Seconds (s)	2.5	2.6	2.0
Stickies	Counts	0	0	0
Burst strength	lb/inch^2^	24.5	22.4	23.3
	Index value	0.213	0.207	0.222
Short Span Compression Strength (STFI)	lb/inch	5.82	5.40	5.52
	Index value	0.051	0.050	0.053

## Conclusions

In summary, we developed a high-barrier and recyclable alternative by coating paper with biodegradable poly­(vinyl alcohol) loaded with laponite and montmorillonite clays. Paper samples coated with PVOH that bear a 2:1 ratio (v/v) of clay to surfactant exhibited enhanced barrier properties, mechanical strength, and thermal stabilities compared to the control samples. Compared to PET film, the laponite and montmorillonite paper samples showed 93% and 82% reductions in their oxygen permeability, respectively. LPD and MCPD papers were found to be repulpable (99%) and recyclable according to TAPPI certified protocols. This work offers a packaging solution in which gas barrier properties, mechanical strength, cost, and environmental safety are crucial. In addition, compared to PET film, the tested samples are potentially more suitable for packaging applications requiring a high oxygen barrier performance.

## Supplementary Material



## Notes

Additional details of the experiments, including: a table showing general details of formulations made and tested for all seven clays (Table S1); a table showing details of laponite formulations (Table S2); a table showing details of montmorillonite formulations (Table S3); OTR data for montmorillonite formulations with single-layer PVOH-coated papers (Table S4); OTR results of montmorillonite formulations with double-layer PVOH-coated papers (Table S5); WVTR data for montmorillonite formulations with single-layer PVOH-coated papers (Table S6); WVTR data for montmorillonite formulations with double-layer PVOH-coated papers (Table S7); OTR data for laponite formulations with single-layer PVOH-coated papers (Table S8); OTR data for laponite formulations with double-layer PVOH-coated papers (Table S9); WVTR data for laponite formulations with single-layer PVOH-coated papers (Table S10); WVTR data for laponite formulations with double-layer PVOH-coated papers (Table S11); a comparison of this work with our previous studies on high-barrier paper packaging (Table S12); clay dispersion series obtained from (a) nanoclay surface modified, (b) montmorillonite, (c) hydrotalcite, (d) kaolin, (e) nanoclay hydrophilic bentonite, (f) laponite, (g) Cellulose Nanocrystal (Figure S1); dispersions obtained from montmorillonite clay (a) and laponite clay (b) at different concentrations (Figure S2); complete (a) TGA and (b) DTG curves of paper samples, including B-KP, P-KP, LPD, and MCPD (Figure S3); SEM images of paper samples coated with clay dispersions prepared in deionized water without LignoSAS surfactant (a) LPA; (b) LPB; (c) MCPA; (d) MCPB; (e) double-layer coated PVOH paper (Figure S4); 100 nm TEM images of LPD (a_1_ and a_2_) and MCPD (b_1_ and b_2_) coatings (Figure S5); the screen accepts obtained for sample LPD (a); screen rejects obtained for sample LPD (b); screen accepts obtained for sample MCPD (c); and screen rejects obtained for sample MCPD (d) (Figure S6); handsheets prepared using pulp from (a) LPD (b) MCPD, both sheets have no stickies on them (Figure S7); SEM Images of (a) the vortex-only laponite dispersion, (b) the sonicated laponite dispersion at lower magnification, (c) the sonicated laponite dispersion at higher magnification, (d) the vortex-only montmorillonite dispersion, (e) the sonicated montmorillonite dispersion at lower magnification (f) the sonicated montmorillonite dispersion at higher magnification (Figure S8); SEM Images of clay dispersions before mixing with PVOH for (a) the nonsonicated laponite clay dispersion (b) the sonicated laponite dispersion, (c) the nonsonicated montmorillonite dispersion and (d) the sonicated montmorillonite dispersion (Figure S9); 100 nm TEM images of (a) sonicated laponite dispersions obtained from four different regions (a1, a2 a3, a4) of the grid (b) sonicated montmorillonite dispersions obtained from four different regions (b1, b2 b3, b4) of the grid (Figure S10); (a) a comparison of the WP of initial paper samples and paper samples coated with the sonicated dispersions at same humidity and temperature conditions (90% RH; 23 °C) (b) Comparison of the OP of initial paper samples and paper samples coated with the sonicated dispersions at same humidity and temperature conditions (50% RH, 23 °C) (Figure S11); DSC thermograms of neat PVOH, dried LPD dispersion and dried MCPD dispersion (Figure S12); (a) oxygen permeabilities (OP) of films of PVOH, LPD, and MCPD coatings, with respect to the blank kraft paper (B-KP). The OP for B-KP was beyond the detection limit (BDL); (b) Water vapor permeabilities (WP) of films of PVOH, LPD, and MCPD coatings, with respect to B-KP (Figure S13) (PDF)

## Notes

Chemistry Department, Faculty of Science, Tanta University, Tanta 31527, Egypt
