# Supplementary material for: Moving Toward Paperization of Packaging Industry: Use of Laponite and Montmorillonite Nanoclays for Recyclable and Biodegradable High-Barrier Paper
Source: ACS Omega. 2026 Feb 3;11(6):9876–89. doi: 10.1021/acsomega.5c10619 (PMC12917786; doi:10.1021/acsomega.5c10619)
Supplement: Supplementary file 1 [file ao5c10619_si_001.pdf]

Supporting Information

For

Moving towards Paperization of Packaging Industry: Use of laponite and montmorillonite nanoclays for recyclable and biodegradable high-barrier paper

Paninga Muiliya[1,2#], Syeda Shamila Hamdani[1#], Mohamed Shaker[1], Ian Wyman[1], and Muhammad Rabnawaz[1,2*]

[1]School of Packaging, Michigan State University, 448 Wilson Road, East Lansing, Michigan, 48824-1223, United States

[2]Department of Chemistry, Michigan State University, East Lansing, Michigan, 48824-1223, United States

*Corresponding author: rabnawaz@msu.edu

[#]Authors with equal contribution

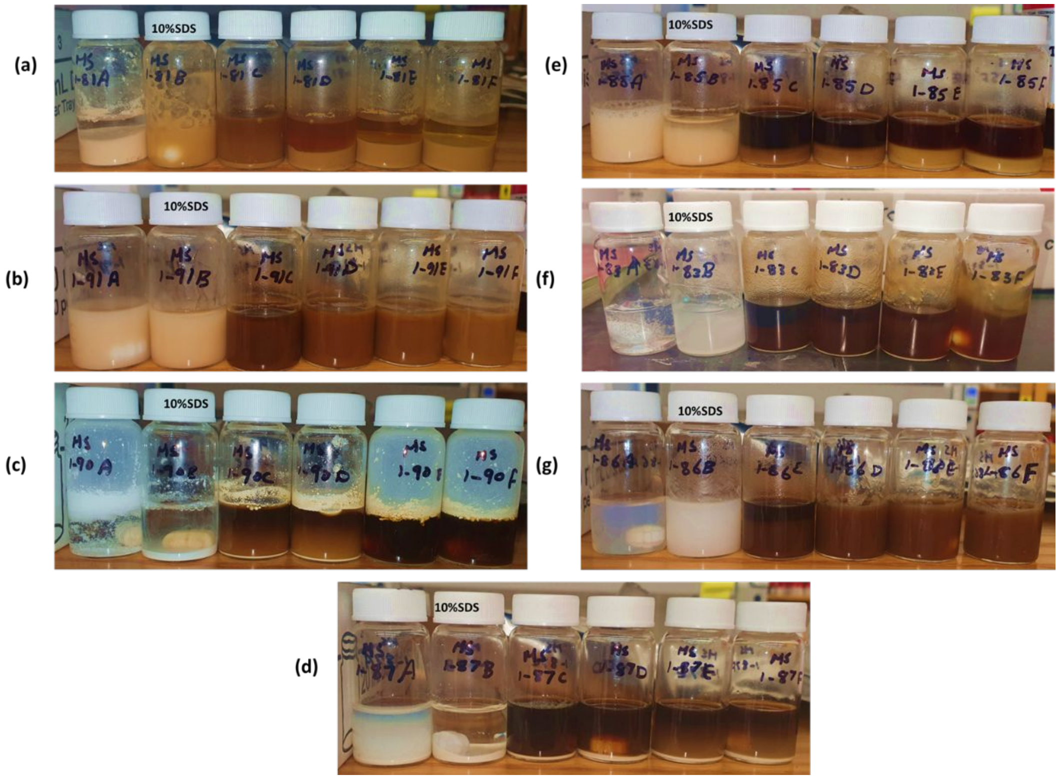

Figure S1: Clay dispersion series obtained from (a) Nano clay Surface Modified, (b) Montmorillonite), (c) Hydrotalcite, (d) Kaolin, (e) Nanoclay hydrophilic bentonite, (f) Laponite, (g) Cellulose Nanocrystal .These clay dispersions are made using the formulation in Table S1

Table S1. Table showing general details of formulations made and tested for all seven clays.

Formulation ID	Nanoclay	LignoSAS	DI water	10 wt. % SDS	Nanoclay: LignoSAS (w/w)	Nanoclay Concentration
CL – A	320 mg	-	8 mL	-	Blank 1	4 wt%
CL – B	320 mg	-	-	8 mL	Blank 2	
CL – C	160 mg	160 mg	8 mL		1:1	
CL – D	214 mg	106 mg	8 mL		2:1	
CL – E	240 mg	80 mg	8 mL		3:1	
CL – F	256 mg	64 mg	8 mL		4:1	

CL – A : Clay formulation with no surfactant, and deionized (DI) water used as solvent.

CL – B: Clay formulation with no surfactant, and 10 wt. % SDS used as solvent.

CL – C: Clay formulation with a 1:1 ratio (w/w) of clay and LignoSAS surfactant.

CL – D: Clay formulation with a 2:1 ratio (w/w) of clay and LignoSAS surfactant.

CL – E: Clay formulation with a 3:1 ratio (w/w) of clay and LignoSAS surfactant.

CL – F: Clay formulation with a 4:1 ratio (w/w) of clay and LignoSAS surfactant.

Where "CL" is clay, including Nano clay Surface Modified, Montmorillonite, Hydrotalcite, Kaolin, Nanoclay hydrophilic bentonite, Laponite, and Cellulose Nanocrystal.

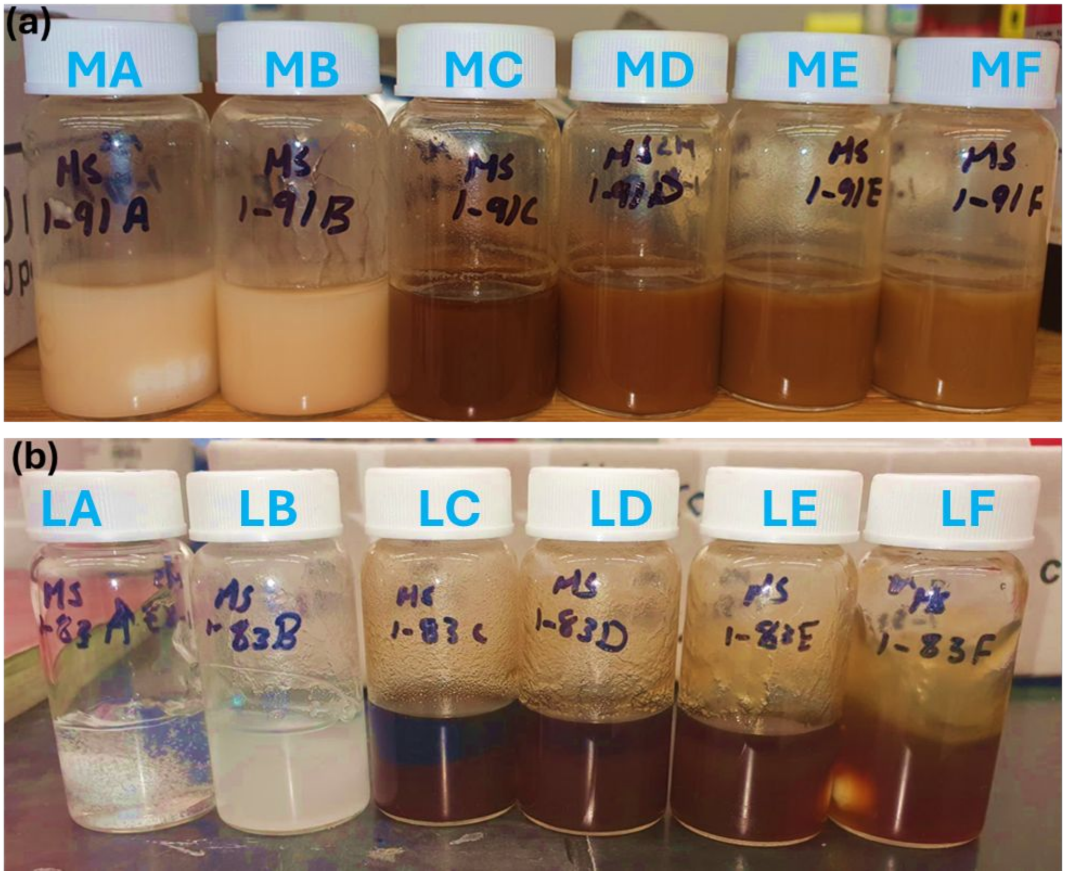

Figure S2. Dispersions obtained from montmorillonite clay **(a)** and laponite clay **(b)** at different concentrations. Refer to Tables **S2 and S3** for formulations of each sample. The two set of clay dispersions were selected based on their superior stability.

Table S2. Table showing details of laponite formulations.

Formulation ID	Laponite	LignoSAS	DI water	10 wt. % SDS	Laponite: LignoSAS (w/w)	Nanoclay Concentration
LA	320 mg	-	8 mL	-	Blank 1	4 wt%
LB	320 mg	-	-	8 mL	Blank 2	
LC	160 mg	160 mg	8 mL		1:1	

LD	214 mg	106 mg	8 mL		2:1	
LE	240 mg	80 mg	8 mL		3:1	
LF	256 mg	64 mg	8 mL		4:1	

LA: Laponite clay formulation with no surfactant, and deionized (DI) water used as solvent.

LB: Laponite clay formulation with no surfactant, and 10 wt. % SDS used as solvent.

LC: Formulation with a 1:1 ratio (w/w) of laponite clay and LignoSAS surfactant.

LD: Formulation with a 2:1 ratio (w/w) of laponite clay and LignoSAS surfactant.

LE: Formulation with a 3:1 ratio (w/w) of laponite clay and LignoSAS surfactant.

LF: Formulation with a 4:1 ratio (w/w) of laponite clay and LignoSAS surfactant.

Table S3. Table showing details of montmorillonite formulations.

Formulation ID	Montmorillonite	LignoSAS	DI water	10 % SDS	Montmorillonite : LignoSAS (w/w)	Nanoclay Concentration
MA	320 mg	-	8 mL	-	Blank 1	4 wt%
MB	320 mg	-	-	8 mL	Blank 2	
MC	160 mg	160 mg	8 mL		1:1	
MD	214 mg	106 mg	8 mL		2:1	
ME	240 mg	80 mg	8 mL		3:1	
MF	256 mg	64 mg	8 mL		4:1	

MA: Montmorillonite clay formulation with no surfactant, and DI water used as solvent.

MB: Montmorillonite clay formulation with no surfactant, and 10wt% SDS used as solvent.

MC: Formulation with a 1:1 ratio of Montmorillonite and LignoSAS surfactant.

MD: Formulation with a 2:1 ratio of Montmorillonite clay and LignoSAS surfactant.

ME: Formulation with a 3:1 ratio of Montmorillonite clay and LignoSAS surfactant.

MF: Formulation with a 4:1 ratio of Montmorillonite clay and LignoSAS surfactant.

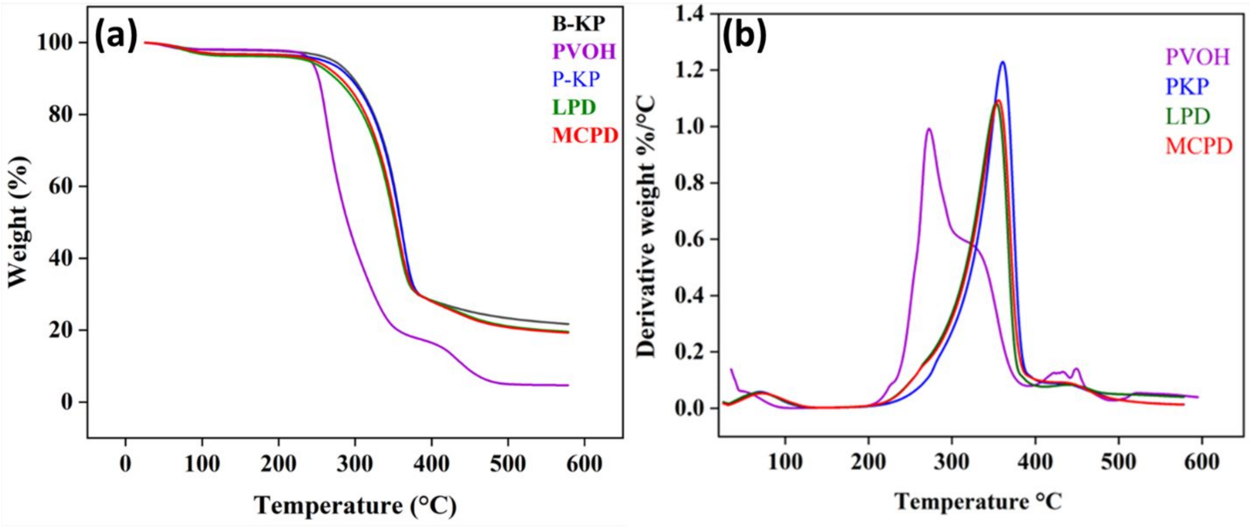

Figure S3. Complete (a) TGA and (b) DTG curves of paper samples including B-KP, P-KP, LPD, and MCPD.

PVOH displayed an intrinsic thermal signature at 230 – 350 ℃ for the pyrolysis of the amorphous region, which is primarily characterized by dehydration [1], [2]. The region around 350 – 460 ℃ is attributed to the degradation of the crystalline region, which then results in the formation of a residual mass of 4.65 %. Kraft paper showed thermal stability around 300 to 400 ℃, with onset at 328 ℃ (Figure S), and forms 21.59 % residue. This attribute is possibly due to the presence of components like cellulose and lignin in the paper[3]. The nanoclay-coated papers showed similar thermal property with no significant improvement as compared to the P-KP sample. Like our previous work on Zein and PVOH[4], this could be due to the low-coating load of the paper samples.

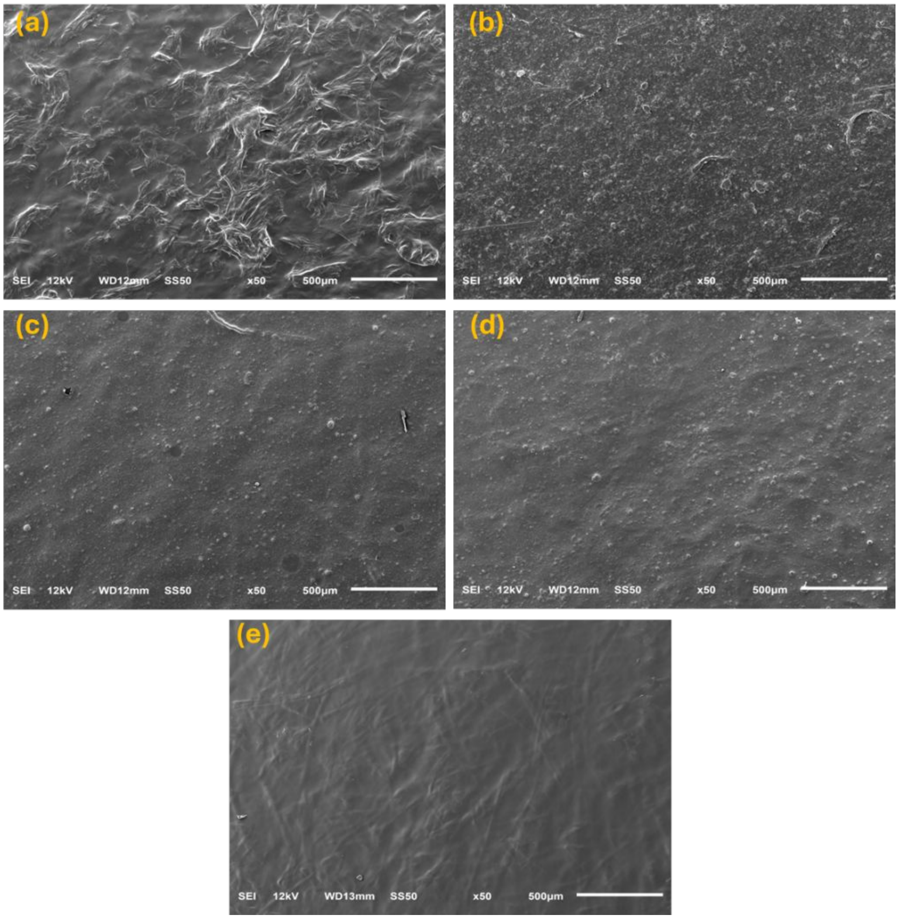

Figure S4. SEM images of paper samples coated with clay dispersions prepared in deionized water without LignoSAS surfactant (a) LPA; (b) LPB; (c) MCPA; (d) MCPB; (e) double-layer coated PVOH paper. See **Table 2** for the description, thickness, basis weight, and coating load values of the paper samples.

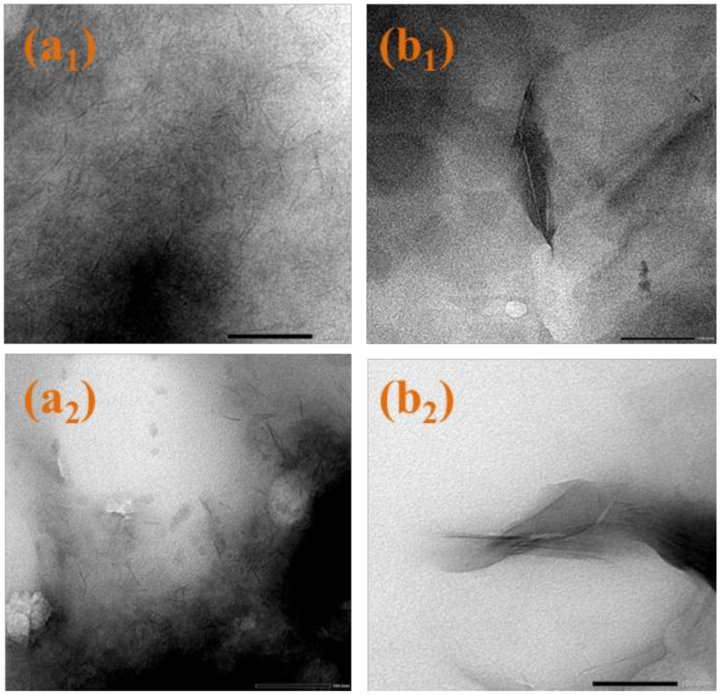

Figure S5. 100 nm TEM images of LPD (a_1 and a_2) and MCPD (b_1 and b_2) coatings. Images were taken at different sites (1 and 2) to observe the distribution and exfoliation of clay particles in the polymer matrix

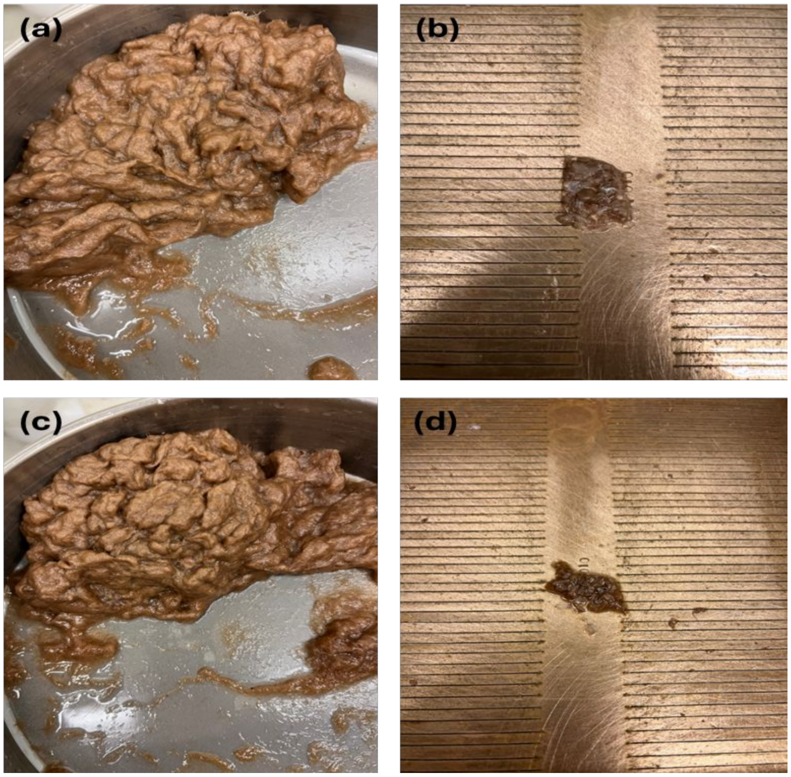

Figure S6. The screen accepts obtained for sample LPD (a), screen rejects obtained for sample LPD (b), screen accepts obtained for sample NCPD (c), and screen rejects obtained for sample NCPD (d).

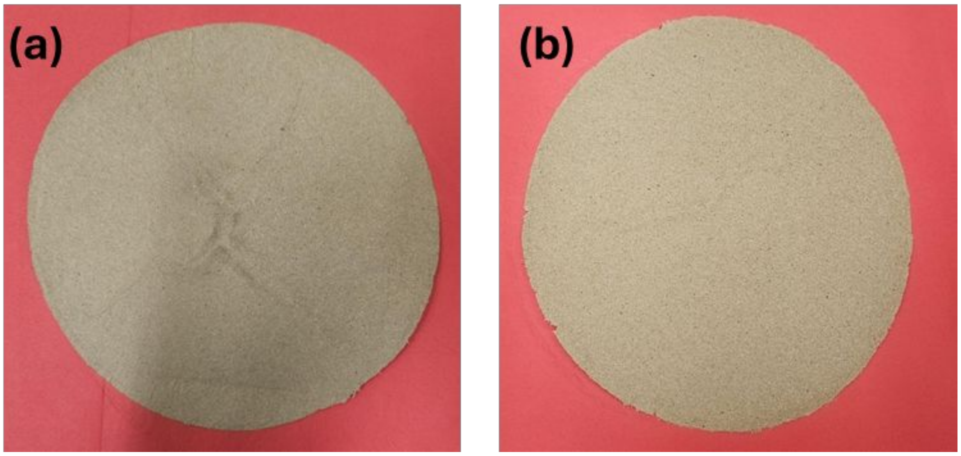

Figure S7. Handsheets prepared using pulp from (a) LPD and (b) NCPD. Both sheets have no stickies on them.

Table S4. OTR data obtained for montmorillonite formulations with single-layer PVOH-coated papers.

S/No	Formulation<br>S- Single layer of PVOH as the base layer	Nanoclay: PVOH ratio (v/v	Oxygen permeation (OP) at 100%-<br>cc · mm/(m² · day)
1	MA – S	1:2	847.05 ± 72.05
		1:1	≥3184.70
2	MB – S	1:2	4.03 ± 1.09
		1:1	≥3184.70
3	MC – S	1:2	158.54± 17.16
		1:1	219.95±56.11
4	MD – S	1:2	2.41 ± 2.24
		1:1	649.50 ± 26.20
5	ME – S	1:2	259.26 ± 83.08
		1:1	2.88 ± 1.40
6	MF – S	1:2	2.79 ± 2.78
		1:1	≥3184.70

Table S5. OTR data obtained for montmorillonite formulations with double-layer PVOH-coated papers.

S/No	Formulation<br>D- Double layer of PVOH as the base layer	(Nanoclay: PVOH ratio (v/v)	Oxygen permeation (OP) at 100%-<br>cc · mm/(m² · day)

1	MA – D	1:2	2.38 ± 0.10
		1:1	246.72 ± 5.30
2	MB – D	1:2	3.92 ± 0.74
		1:1	8.78 ± 3.32
3	MC – D	1:2	6.33 ± 6.02
		1:1	4.66 ± 3.16
4	MD – D	1:2	1.95 ± 0.80
		1:1	4.87 ± 1.56
5	ME – D	1:2	1.35 ± 0.44
		1:1	
6	MF – D	1:2	3.08 ± 0.46
		1:1	239.20±6.40

Table S6. WVTR data obtained for montmorillonite formulations with single-layer PVOH-coated papers.

S/No	Formulation<br>S- Single layer of PVOH as the base layer	Nanoclay: PVOH ratio (v/v)	Permeation (WP) at 90% g·mm/(m² · day)
1	MA – S	1:2	38.96±4.71
		1:1	33.39±1.14
2	MB – S	1:2	28.57±4.93
		1:1	30.75±0.55
3	MC – S	1:2	46.07±1.27
		1:1	51.39±1.75
4	MD – S	1:2	48.56±2.29
		1:1	48.35±2.31

5	ME – S	1:2	44.82±3.48
		1:1	48.26±1.21
6	MF – S	1:2	41.86±3.00
		1:1	44.88±5.32

Table S7. WVTR data obtained for montmorillonite formulations with double-layer PVOH-coated papers.

S/No	Formulation<br>D- Double layer of PVOH as the base layer	Nanoclay: PVOH ratio (v/v)	Permeation (WP) at 90% g·mm/(m^2 · day)
1	MA – D	1:2	14.58±1.07
		1:1	14.00±1.56
2	MB – D	1:2	37.10±0.29
		1:1	35.17±1.57
3	MC – D	1:2	37.09±0.75
		1:1	36.05±0.01
4	MD – D	1:2	40.28±0.16
		1:1	38.44±1.17
5	ME –D	1:2	35.02±0.19
		1:1	37.79±4.96
6	MF – D	1:2	35.90±0.11
		1:1	48.04±7.40

Table S8. OTR data obtained for laponite formulations with single-layer PVOH-coated papers.

S/No	Formulation<br>S- Single layer of PVOH as the base layer	Nanoclay: PVOH ratio (v/v)	Permeation (OP) at 100%- cc · mm/(m² · day)
1	LA – S	1:2	444.51±6.88
		1:1	136.41±74.31
2	LB – S	1:2	≥3184.70
		1:1	9.99±6.09
3	LC – S	1:2	8.45±2.77
		1:1	3.36±1.07
4	LD – S	1:2	3.79±0.41
		1:1	14.94±8.58
5	LE – S	1:2	4.90±0.66
		1:1	1.45±1.29
6	LF – S	1:2	0.43±0.30
		1:1 S	-

Table S9. OTR data obtained for laponite formulations with double-layer PVOH-coated papers.

S/No	Formulation	Nanoclay: PVOH ratio (v/v)<br>D- Double layer of PVOH as the base layer	Permeation (OP) at 100%-<br>cc · mm/(m² · day)
1	LA – D	1:2	632.79±17.65
		1:1	1.37±0.86
2	LB – D	1:2	3.17±3.28
		1:1	0.81±0.41
3	LC – D	1:2	1.37±0.32
		1:1	12.44±0.76
4	LD – D	1:2	0.88±0.02
		1:1	0.81±0.41
5	LE – D	1:2	1.19±0.23
		1:1	2.99±1.23
6	LF – D	1:2	1.93±0.08
		1:1	1.69±0.38

Table S10. WVTR data obtained for laponite formulations with single-layer PVOH-coated papers.

S/No	Formulation<br>S- Single layer of PVOH as the base layer	Nanoclay: PVOH ratio (v/v)	Permeation (WP) at 90%<br>g·mm/(m^2 · day)
1	LA – S	1:2	46.69±1.09
		1:1	46.41±3.99
2	LB – S	1:2	36.97±1.48
		1:1	31.13±3.01
3	LC – S	1:2	43.59±0.59
		1:1	50.32±0.44
4	LD – S	1:2	42.85±3.85
		1:1	47.34±3.03
5	LE – S	1:2	42.18±3.91
		1:1	45.85±5.97
6	LF – S	1:2	47.15±2.13
		1:1	48.99±0.37

Table S11. WVTR data obtained for laponite formulations with double-layer PVOH-coated papers.

S/No	Formulation<br>D- Single layer of PVOH as the base layer	Nanoclay: PVOH ratio (v/v)	Permeation (WP) at 90%<br>g·mm/(m² · day)
1	LA – D	1:2	43.43±1.31
		1:1	38.97±5.04
2	LB – D	1:2	32.72±4.21
		1:1	34.61±0.54
3	LC – D	1:2	38.16±9.05
		1:1	43.85±1.75
4	LD – D	1:2	37.23±3.71
		1:1	34.77±6.39
5	LE – D	1:2	38.96±6.68
		1:1	40.83±2.77
6	LF – D	1:2	34.38±1.76
		1:1	40.00±2.19

Table S12: Comparison of this work with our previous studies on high-barrier paper packaging.

S/N	Sample	WVTR (g/m² · day)	WP g·mm/m² · day)	OTR (cc/m² · day)	OP cc·mm/m²·day)	Ref
1	LPD	187.985±18.74	34.12±2.34	4.145±0.08	0.875 ± 0.02	This work
2	MCPD	196.481±0.78	40.28 ± 0.16	9.515±3.92	1.95± 0.81	This work

3	P5Z12.5-18 pt	-	-	128.0 ± 14.7	*66.56	[5]
4	SZG20-D	299.2 ± 18.6	*218.42	262.31 ± 10.66	*191.48	[6]

*Normalized values of the OTR and WVTR of the samples were calculated by multiplying the transmission values by the estimated value of thickness from the OTR and WVTR graphs. All OTR analyses reported in the table were conducted at 23°C and 50 % relative humidity.

Effect of Sonication on Clay dispersion and morphology.

We also evaluated the effect of sonication on the particle size, coating morphology and barrier properties of resultant coated papers. The polymer-clay dispersions of the best performing samples, LPD and MCPD, were sonicated for 3 h. SEM and TEM analyses were conducted on these sonicated dispersions and compared with vortex-only dispersions (without sonification), as presented in (a) and (b) below. SEM and TEM analyses were also conducted on the clay dispersed in water only, both with sonication and without sonication.

a. SEM Analysis of Clay Dispersions

At lower magnification, the sonicated laponite (LPD) polymer-clay dispersion (**Figure S8b**) showed rounded clusters that resemble nanoclay tactoids, while the sonicated montmorillonite (MCPD) dispersion (**Figure S8e**) showed a finer particle distribution. Images at higher magnification were taken and compared with high magnification images of the vortex-only dispersion. The sonicated laponite dispersion (**Figure S8c**) showed fragmented clay aggregates with slightly larger sizes than those of the vortex-only dispersion **(Figure S8a)**, which could be a result of re-aggregation of the platelets. Little change was observed in the sonicated montmorillonite dispersion (**Figure S8f**), as compared to the vortex-only dispersion (**Figure S8d).** This shows that no significant change in particle size was observed after sonication. Therefore, we proceeded without sonication, as it does not substantially impact on the dispersion quality.

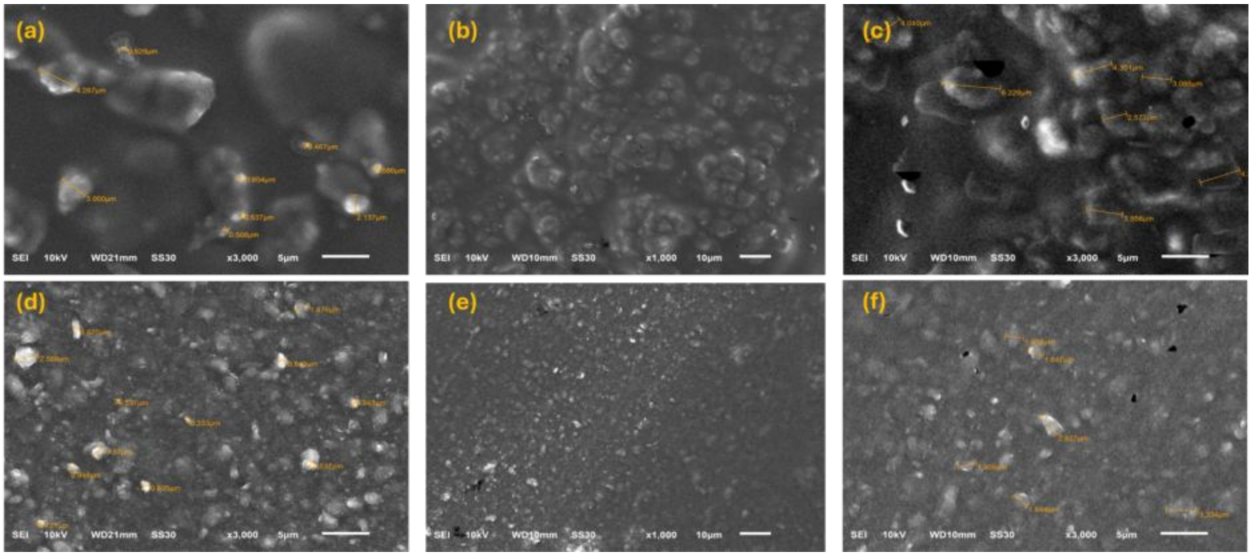

Figure S8. SEM Images of (a) the vortex-only laponite dispersion, (b) the sonicated laponite dispersion at lower magnification, (c) the sonicated laponite dispersion at higher magnification, (d) the vortex-only montmorillonite dispersion, (e) the sonicated montmorillonite dispersion at lower magnification (f) the sonicated montmorillonite dispersion at higher magnification.

To further evaluate the effect of sonication on the coating, we first dispersed clay and LignoSAS in water only and stirred for 3 h, in accordance with the protocol reported in the main article. This was then sonicated for 3 h. The SEM of the sonicated clay dispersion was measured and compared it with the clay dispersion without sonication (**Figure S9a**). The initial uniformity of laponite dispersion appeared to be disrupted (**Figure S9b**), possibly due to reaggregation of the particles due to increased mixing. Little change was observed in the morphology of the sonicated montmorillonite dispersion (**Figure S9d**).

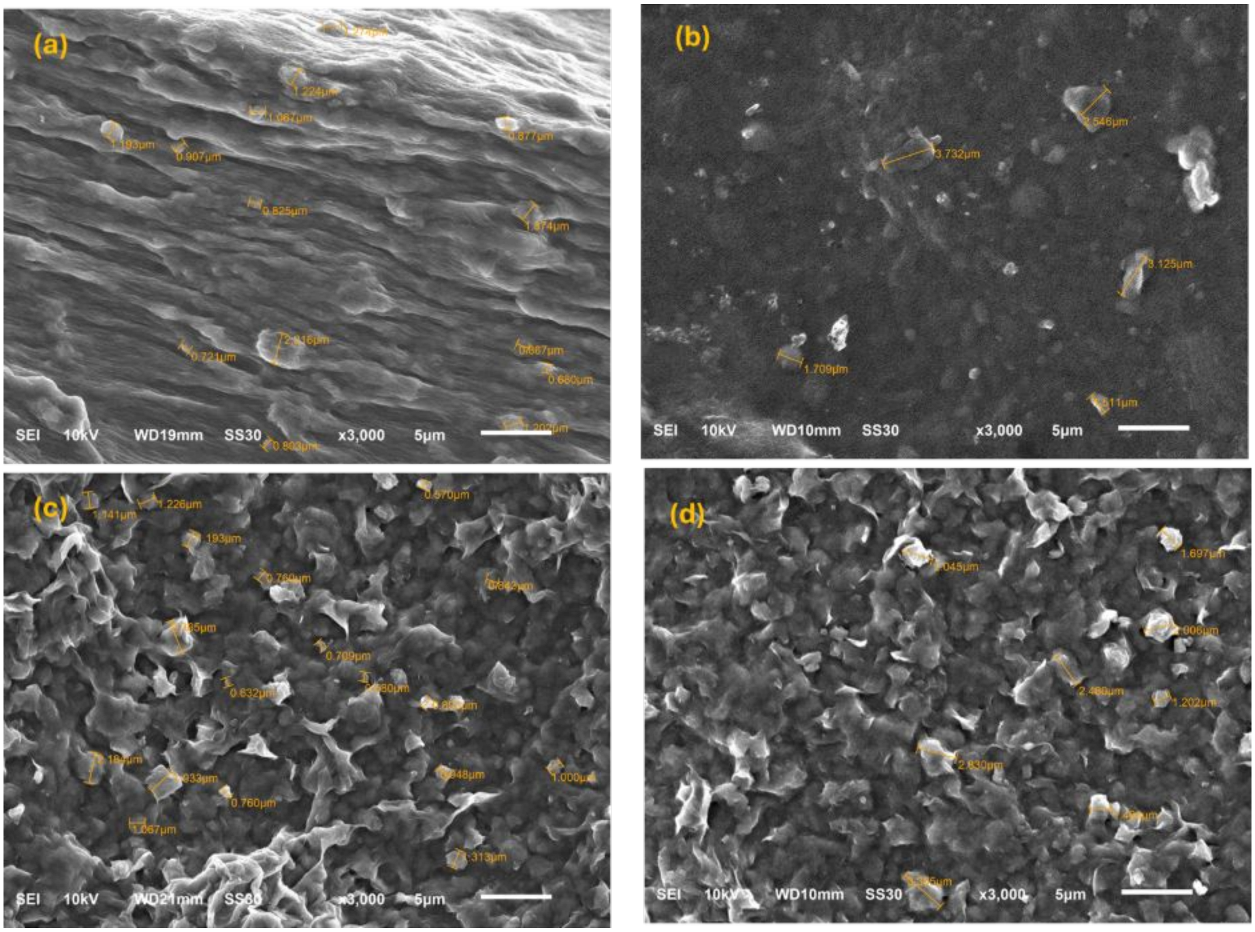

Figure S9: SEM Images of clay dispersions before mixing with PVOH for (a) the non-sonicated laponite clay dispersion (b) the sonicated laponite dispersion, (c) the non-sonicated montmorillonite dispersion and (d) the sonicated montmorillonite dispersion.

b. TEM of clay dispersions

The TEM micrographs reveal mixed morphologies in the sonicated LPD and MCPD dispersions, comprising exfoliated platelets, intercalated stacks, and residual aggregates. Relative to the vortex-only dispersion (**Figure S5**), the sonicated sample displays a higher prevalence of intercalated structures. However, the morphological heterogeneity and persistence of aggregation are expected to diminish tortuosity-driven barrier enhancement and thereby temper the overall barrier performance.

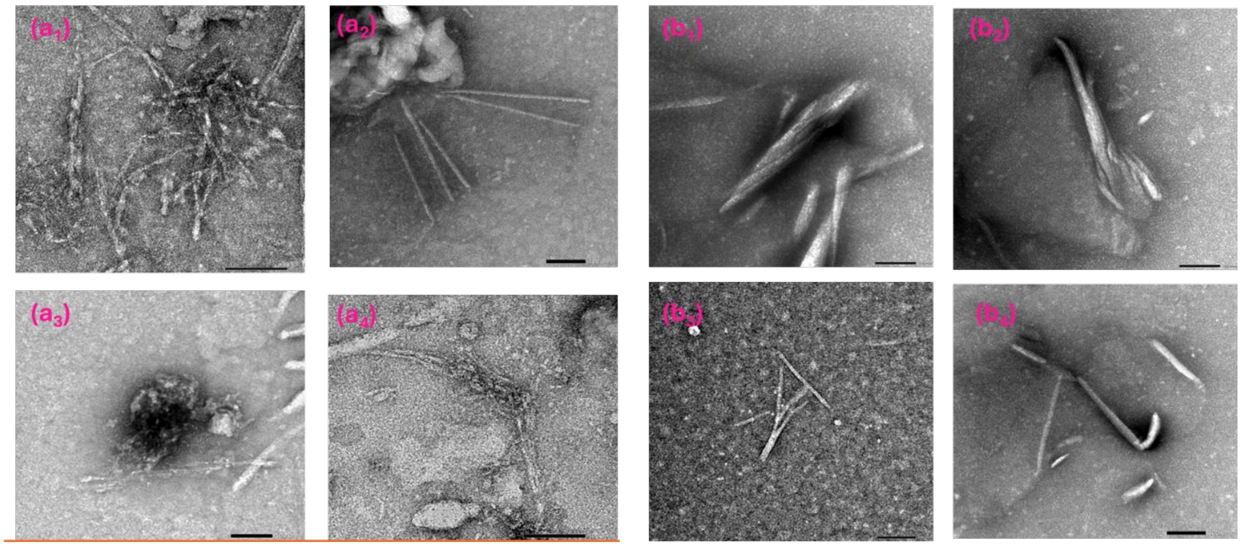

Figure S10. 100 nm TEM images of (a) sonicated laponite dispersions obtained from four different regions (a_1, a_2 a_3, a_4) of the grid (b) sonicated montmorillonite dispersions obtained from four different regions (b_1, b_2 b_3, b_4) of the grid.

Effect of Sonication on Barrier Properties

To explore the effect of sonication on WVTR and OTR, the kraft paper was coated in similar way as it was coated for original dispersions. The results were compared with the barrier properties of the original samples (prepared by applying coating without sonication), as shown in **Figure S11**, where LPD-Son and MCPD-Son represent the paper samples coated with the sonicated LPD and MCPD polymer-clay dispersions. The result shows that sonication has increased the water permeability (WP) of the laponite coated paper slightly from 35.59±0.25 (LPD) to 46.58±4.29 g ·mm/m² · day (LPD-Son.). On the other hand, there was a slight reduction in WP of the MPCD-Son up to 30.72±0.26 g ·mm/m² · day in contrast to the vortex-only MCPD paper sample (40.28±0.16 g ·mm/m² · day).

Similar to WP, the oxygen permeability (OP) of the laponite coated paper slightly increased, from 0.875±0.02 (LPD) to 2.94±0.06 (LPD-Son.) cc.mm/m² · day. The value of the OP of the MCPD-Son paper was not significantly different from that MCPD. Overall, sonification had little or no positive effect on barrier properties of the paper samples.

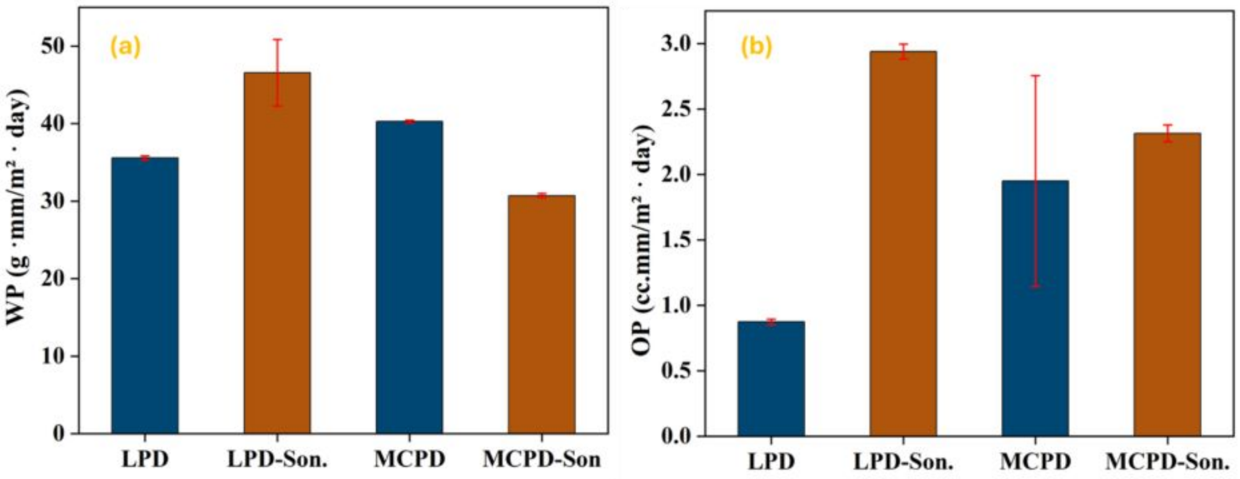

Figure S11. (a) Comparison of the WP of paper samples coated with vortex-only clay dispersions and paper samples coated with the sonicated dispersions at same humidity and temperature conditions (90 % RH; 23 °C) (b) Comparison of the OP of vortex-only paper samples and paper samples coated with the sonicated dispersions at same humidity and temperature conditions (50 % RH, 23 °C)

Differential Scanning Calorimetry

DSC analysis was carried out for three samples—neat PVOH, dried LPD, and MCPD—shown in **Figure S12** . The DSC analysis revealed that neat PVOH displayed a sharp melting endotherm at ~225 °C along with a crystalline domain, confirming its significant crystalline nature.

The elimination of the PVOH endothermic melting and crystallization exotherm peaks in LPD and MCPD is indicative of significant restriction of the polymer chain mobility in polymer clay systems[7]. The DSC thermograms of the PVOH–nanoclay composites (LPD and MCPD) showed reduced melting enthalpies and broad high-temperature endothermic features. We attribute this effect to reduced crystal growth and the endothermic desorption of the strongly bound interlayer water within the polymer clay network[8]. This polymer- clay interaction, resulting in suppressed crystallinity of neat PVOH, is consistent with the mechanism of tortuosity for gas transport in platelet-filled systems [7], [9]

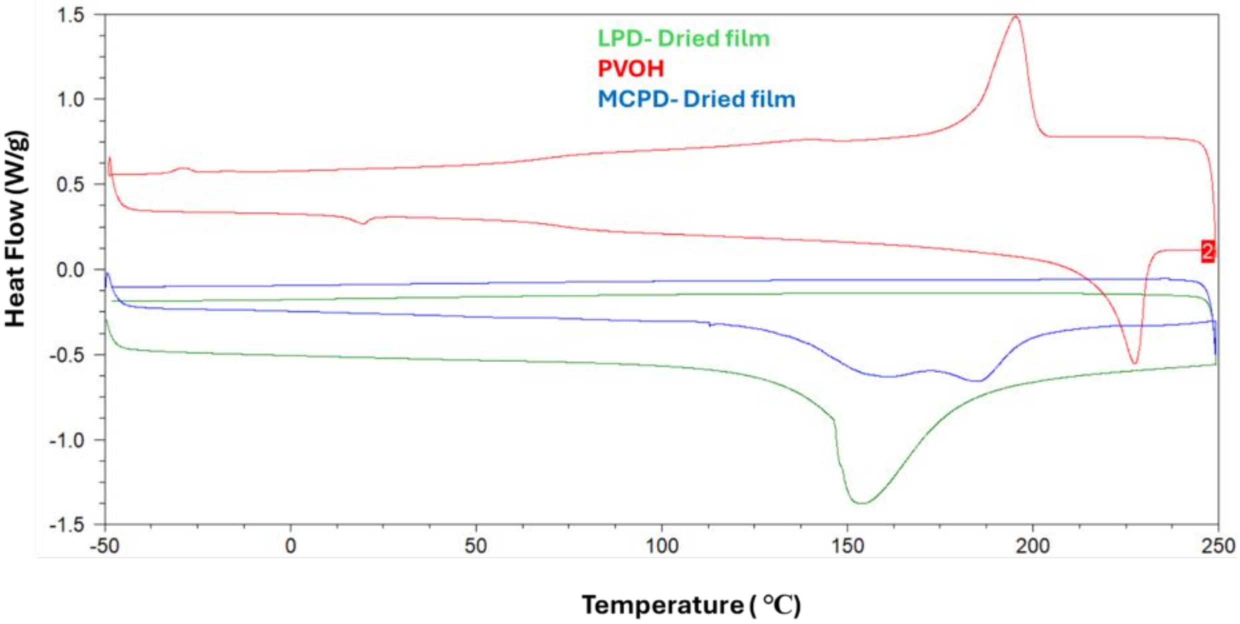

Figure S12. DSC thermograms of neat PVOH, dried LPD dispersion and dried MCPD dispersion.

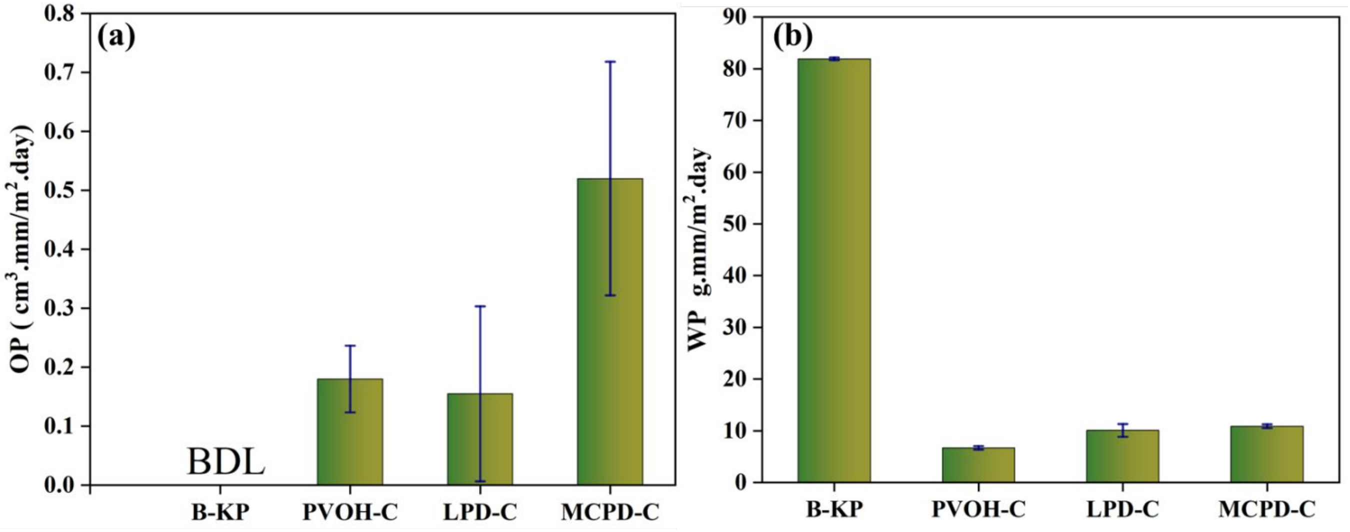

Figure S13. (a) Oxygen permeabilities (OP) of films of PVOH, LPD, and MCPD coatings, with respect to the blank kraft paper (B-KP). The OP for B-KP was beyond detection limit (BDL). (b) Water vapor permeabilities (WP) of films of PVOH, LPD, and MCPD coatings, with respect to B-KP. The coating films have been distinguished from the paper samples by attaching the letter "C' to the abbreviations. The thickness of each film was calculated by subtracting the thickness of the B-KP from the thickness of the dried coated paper (e.g. LPD – BKP). This thickness was then multiplied by gas transmission rate of the paper samples to get the permeation coefficients of the films.

Reference

[1] B. J. Holland and J. N. Hay, "The thermal degradation of poly(vinyl alcohol)," *Polymer (Guildf)*, vol. 42, no. 16, pp. 6775–6783, Jul. 2001, doi: 10.1016/S0032-3861(01)00166-5.

[2] J.-L. Shie, Y.-H. Chen, C.-Y. Chang, J.-P. Lin, D.-J. Lee, and C.-H. Wu, "Thermal Pyrolysis of Poly(vinyl alcohol) and Its Major Products," *Energy & Fuels*, vol. 16, no. 1, pp. 109–118, Jan. 2002, doi: 10.1021/ef010082s.

[3] S. Soares, G. Camino, and S. Levchik, "Comparative study of the thermal decomposition of pure cellulose and pulp paper," *Polym Degrad Stab*, vol. 49, no. 2, pp. 275–283, Jan. 1995, doi: 10.1016/0141-3910(95)87009-1.

[4] S. S. Hamdani, Z. Li, N. Sirinakbumrung, and M. Rabnawaz, "Zein and PVOH-Based Bilayer Approach for Plastic-Free, Repulpable and Biodegradable Oil- and Water-Resistant Paper as a Replacement for Single-Use Plastics," *Ind Eng Chem Res*, vol. 59, no. 40, pp. 17856–17866, Oct. 2020, doi: 10.1021/acs.iecr.0c02967.

[5] S. S. Hamdani, Z. Li, P. Ruoqi, E. Rollend, and M. Rabnawaz, "Oxygen and water vapor barrier properties of polyvinyl alcohol and zein bilayer-coated paper," *J Appl Polym Sci*, vol. 139, no. 7, Feb. 2022, doi: 10.1002/app.51707.

[6] S. S. Hamdani, Z. Li, E. Rolland, M. Mohiuddin, and M. Rabnawaz, "Barrier and mechanical properties of biodegradable paper bilayer-coated with plasticized starch and zein," *J Appl Polym Sci*, vol. 140, no. 8, Feb. 2023, doi: 10.1002/app.53440.

[7] M. Alexandre and P. Dubois, "Polymer-layered silicate nanocomposites: preparation, properties and uses of a new class of materials," *Materials Science and Engineering: R: Reports*, vol. 28, no. 1–2, pp. 1–63, Jun. 2000, doi: 10.1016/S0927-796X(00)00012-7.

[8] K. E. Strawhecker and E. Manias, "Structure and Properties of Poly(vinyl alcohol)/Na $^{+}$ Montmorillonite Nanocomposites," *Chemistry of Materials*, vol. 12, no. 10, pp. 2943–2949, Oct. 2000, doi: 10.1021/cm000506g.

[9] R. K. Bharadwaj, "Modeling the Barrier Properties of Polymer-Layered Silicate Nanocomposites," *Macromolecules*, vol. 34, no. 26, pp. 9189–9192, Dec. 2001, doi: 10.1021/ma010780b.
